# A Compact Representation of Drawing Movements with Sequences of Parabolic Primitives

**DOI:** 10.1371/journal.pcbi.1000427

**Published:** 2009-07-03

**Authors:** Felix Polyakov, Rotem Drori, Yoram Ben-Shaul, Moshe Abeles, Tamar Flash

**Affiliations:** 1 Department of Computer Science and Applied Mathematics, Weizmann Institute of Science, Rehovot, Israel; 2 Department of Physiology, Hadassah Medical School, Hebrew University, Jerusalem, Israel; 3 Gonda Brain Research Center, Bar-Ilan University, Ramat-Gan, Israel; University of Freiburg, Germany

## Abstract

Some studies suggest that complex arm movements in humans and monkeys may optimize several objective functions, while others claim that arm movements satisfy geometric constraints and are composed of elementary components. However, the ability to unify different constraints has remained an open question. The criterion for a maximally smooth (minimizing jerk) motion is satisfied for parabolic trajectories having constant equi-affine speed, which thus comply with the geometric constraint known as the two-thirds power law. Here we empirically test the hypothesis that parabolic segments provide a compact representation of spontaneous drawing movements. Monkey scribblings performed during a period of practice were recorded. Practiced hand paths could be approximated well by relatively long parabolic segments. Following practice, the orientations and spatial locations of the fitted parabolic segments could be drawn from only 2–4 clusters, and there was less discrepancy between the fitted parabolic segments and the executed paths. This enabled us to show that well-practiced spontaneous scribbling movements can be represented as sequences (“words”) of a small number of elementary parabolic primitives (“letters”). A movement primitive can be defined as a movement entity that cannot be intentionally stopped before its completion. We found that in a well-trained monkey a movement was usually decelerated after receiving a reward, but it stopped only after the completion of a sequence composed of several parabolic segments. Piece-wise parabolic segments can be generated by applying affine geometric transformations to a single parabolic template. Thus, complex movements might be constructed by applying sequences of suitable geometric transformations to a few templates. Our findings therefore suggest that the motor system aims at achieving more parsimonious internal representations through practice, that parabolas serve as geometric primitives and that non-Euclidean variables are employed in internal movement representations (due to the special role of parabolas in equi-affine geometry).

## Author Summary

Although our movements are flexible and versatile, they are nonetheless highly stereotypical. This versatility is similar to that of natural language sentences, which are composed of words which, in turn, are constructed from a small alphabet of elementary phonemes. Parabolic drawings are simple, smooth and remain parabolic even when undergoing a specific kind of geometric transformations. Smoothness, invariance and compactness of representation are important in motion planning and in visual feedback processing. Hence stereotypical parabolic sub-movements may serve as appropriate building blocks of complex movements. Given the similarities between motor organization in monkeys and humans and the greater opportunity to record brain activities in monkeys here we study the spontaneous emergence of stereotypical arm movements in monkeys following practice. We show that practice has indeed led to the emergence of a small alphabet of parabolic elements during spontaneous drawing movements. We further use this alphabet to study sequences of parabolic sub-movements with respect to possible decisions concerning the animal's choice of what elements to concatenate into words and sentences. We also propose that the relative simplicity of movement data compared, for example, to acoustic or semantic data makes their analysis a useful tool in studies of binding and cognitive processing.

## Introduction

Despite decades of research on the formation of human hand trajectories, the basic mechanisms of neuromotor control underlying the generation of even the simplest drawing movements remain poorly understood [1]. Various studies have proposed that human movement preparation aims at optimizing either kinematic [2]–[4] or dynamic [5] criteria, or minimizing movement variance [6]–[9]. Studies in vertebrates have suggested that voluntary movements are composed of basic movement elements combined in parallel or sequentially [10]–[17]. Such modular organization can account for the versatility of animal and human movements and for their ability to acquire new skills.

Geometrically invariant properties of drawing movements were formalized by the two-thirds power law [18]. These kinematic constraints were shown to hold both with respect to movement production [19] and perception [20],[21]. Earlier studies also showed that the two-thirds power law is equivalent to moving at a constant equi-affine speed [22]–[24] and there is psychophysical and neurophysiological evidence for the significant role of the invariance of human motion with respect to equi-affine transformations [25]–[27]. We argue that geometric invariance may provide a more compact representation of complex movements composed of geometric primitives.

Straight point-to-point movements show geometric invariance under dynamic perturbations involving the use of either elastic or viscous loads [15],[28]. Point-to-point movements retain the invariance of their geometric properties even when subjects are required to control the movements of a cursor on a computer screen by moving their fingers in an instrumented data glove [29]. Recent studies in monkeys [25],[27],[30] and humans [31] have indicated that repeatable geometric (curved) shapes used in the construction of complex trajectories emerge after extensive practice in the generation of drawing and sequential movements.

The ability to unify different kinds of movement constraints (optimality, compositionality, geometric invariance) in the modeling of human and animal movements could lead to further insights [4],[27]. Parabolic movement primitives meet the demands of geometric invariance, kinematic optimality of movements and simplicity of movement representation, and may subserve as underlying building blocks in arm trajectory formation [25],[27]. Here, the hypothesis that parabolic segments are geometric primitives in practiced movements was experimentally tested using spontaneous scribbling movements made by two monkeys. Our choice of the source of the data (studying monkey rather than human drawings) was motivated by the feasibility of subsequently analyzing the underlying activity of motor cortical neurons [27].

The predictions of both the two-thirds power law [18] and the constrained minimum-jerk model [4] are identical for a single parabolic stroke [27],[30]. The fit of the recorded trajectories to the predictions of these two models was assessed (based on modeling and analysis of equi-affine speed) and is described in detail in Text S1. Preliminary version of our findings was presented at the Tenth Biennial Conference of the International Graphonomics Society in 2001 (URL of the proceedings paper: http://www.wisdom.weizmann.ac.il/~felix/texts/IGS2001.pdf) and at the Computational Motor Control Workshops at Ben-Gurion University in 2005 and 2006.

## Methods

### Ethics statement

All animals were handled in strict accordance with good animal practice as defined by the relevant national and local animal welfare bodies, and all animal work was approved by the appropriate committee Permit No. OPRR-A01-5011.

### Brief overview of equi-affine invariance

Parabolas play a special role in equi-affine geometry and motor control [27]. In particular, parabolas are the only equi-affine invariant curves for which predictions of the constrained minimum-jerk and the two-thirds power law coincide. Equi-affine invariant curves are considered because of the importance of equi-affine invariance in both human production and perception. Equi-affine transformations of curves differ from the widely known Euclidian transformations. Euclidian transformations preserve distances, whereas equi-affine transformations preserve only areas and parallelism of lines.

Two important equi-affine invariant parameters are equi-affine length (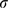) and equi-affine curvature. Time derivative of the equi-affine length of the trajectory (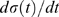) called equi-affine velocity is exactly the piece-wise constant velocity gain factor from the two-thirds power law relating movement speed and Euclidian curvature [22],[24]. Equi-affine transformations constitute the largest subgroup of affine geometric transformations that preserves the velocity gain factor of the two-thirds power law. Equi-affine curvature can be used to classify curves in equi-affine geometry: whenever two curves have the same equi-affine curvature, one curve can be obtained from the other by applying a unique equi-affine transformation. Parabolas have zero equi-affine curvature; therefore any two parabolic segments can be aligned by some affine transformation [27]. The notions of equi-affine geometry and the rationale for its application in motor control studies are described elsewhere [22]–[25],[27],[32],[33]. We provide essential definitions, explanations, and methods of analysis of movements' kinematic parameters in the framework of equi-affine geometry in Text S1.

### The behavioral task and data acquisition

**Figure 1:**
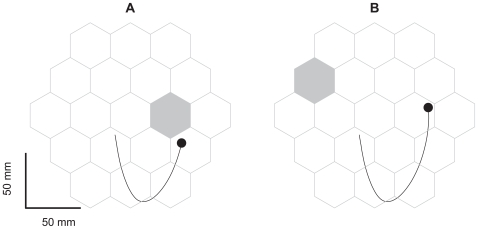
The behavioral procedure used with monkey U. Shown are the grid of possible targets (monkey O had an equivalent grid of circles) and an example of a scribbling movement produced by the monkey. The grey hexagons indicate the single currently active targets. Both the trajectory and the grid were invisible to the monkey. The only visual feedback was produced by the cursor (circle), which indicated the online hand position. A. The monkey's hand is near the target. B. As soon as the monkey's hand entered the target, a beep was heard, the monkey received a little orange juice reinforcement, and another target was randomly selected.

The subjects in this study were two monkeys, O and U (female Macaca fascicularis, 2.6/3.5 kg, respectively). Animal handling procedures conformed to the NIH Guide for the Care and Use of Laboratory Animals (1996), complied with Israeli law, and were approved by the Ethics Committee of the Hebrew University. During the experiments, each monkey sat in a primate chair with the left hand restrained and the right hand operating a two-joint low-friction manipulandum. Following a period of practice, the monkeys created smooth and continuous scribbling movements (Figure 1A). During the entire recording session the monkeys saw nothing but a circular cursor (diameter: 10/4 mm, monkey O/U respectively) indicating the position of the hand.

To motivate the monkey to generate continuous scribbling movements, the working plane was tiled with a grid of 19 possible targets (monkey O circles with radius of 20 mm, monkey U hexagons with edge length of 20 mm). At the beginning of each session, a single target was randomly chosen. As soon as the cursor entered this invisible target, a short beep was produced, and a juice reward was released and delivered for 50 msec. The beep comes with the valve's release of the juice, but it takes a while before the juice actually starts dripping from the spout. We found that when the monkey does not protrude its lips and starts licking the juice spout, much of the reward-juice is spilled down creating a sticky mess on both the monkey's fur and the monkey's chair.

Following a successful hit, another target (also invisible) was randomly selected. Whenever the monkey did not succeed in locating the target within 5 seconds, the target was randomly changed.

The monkey had no knowledge of target location. It adopted a strategy of producing trajectories that covered the entire workspace. The monkey was not required to stop at any stage of the experiment. Figure 1 depicts the grid and task sequence. In a typical session, the monkeys worked for 1.5–2 hours and received 800–1500 rewards. During the first 4 days of practice for both monkeys, the average inter-reward time intervals within each session decreased from 4–5 seconds to 2–3 seconds simultaneously with the increase in the speed of drawing. There was nothing in the training to hint to the monkey that it should search for the target.

The total length of monkey's U arm including the upper arm and forearm segments was 215 mm, and the length of its open hand was 75 mm; the corresponding measurements for monkey O were almost the same. The diameter of the working area was approximately 173 mm (Figure 1A). Therefore, the production of hand movements within the workspace demanded movements of the shoulder and elbow. Hence, the monkeys indeed operated the manipulandum by moving their limbs and not only through wrist rotations (power grip movements).

Hand position in the two dimensional plane was sampled at 100 Hz and logged on a custom-designed data acquisition system. Coordinate data were smoothed using a Gaussian filter with a low-pass cutoff frequency of 8 Hz. Velocity, acceleration and jerk of the hand coordinates were estimated using finite difference approximations of the first- second- and third-order derivatives of position with respect to time:
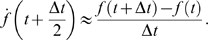



The equi-affine parameters (equi-affine velocity 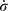 and curvature 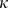) were numerically estimated using a geometrical approximation method [34] which is based on fitting the position data with conics at 5 consecutive data points along the measured path.

To analyze the strategy of the monkeys' drawings, we applied the notion of dwell distribution for the endpoint of the manipulandum position. It is defined here as the frequencies of visiting small parts of the workspace weighted by the movement tangential velocity. That is, visiting some location once with a tangential velocity of 450 mm/s makes the same contribution as visiting the same location 3 times with a tangential velocity of 150 mm/s. This weighting helps to avoid high contributions of slow movements or of periods of rest in the dwell distributions.

### Segments of motion and rest

The monkeys spontaneously switched between periods of rest, with no or very slow motion, and periods of active drawing. We analyzed data from movement segments of the drawings detected by the following procedure:

1. Segments during which the tangential velocity was above a threshold of 150 mm/s were detected.
2. Rest was defined as those portions of the trajectory whose tangential velocity was slower than the threshold for at least 0.2 s. Segments of active motion separated by segments of rest were then identified.
3. The identified movement segments were prolonged for 0.1 s. forwards and backwards in time, or to the closest minima in the tangential velocity, whichever came first.

**Figure 2:**
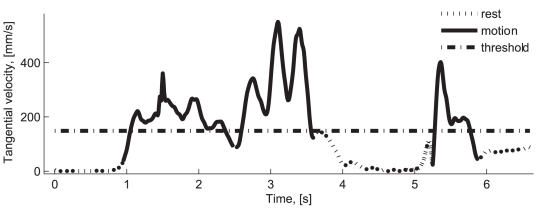
Segments of motion and rest. Three movement segments and four intervals of rest.

An example of the tangential velocity profile for 3 movement segments is shown in Figure 2. The least number of movement segments (57) was registered for monkey U's first practice session. After a period of practice, at least 500 movement segments were typically obtained from a recording session. It should be noted that movement segments are identified based on the values of the tangential velocity of the spontaneously generated movements and that the segmentation procedure did not consider rewarding the monkeys.

### Fitting drawings with parabolic segments

**Figure 3:**
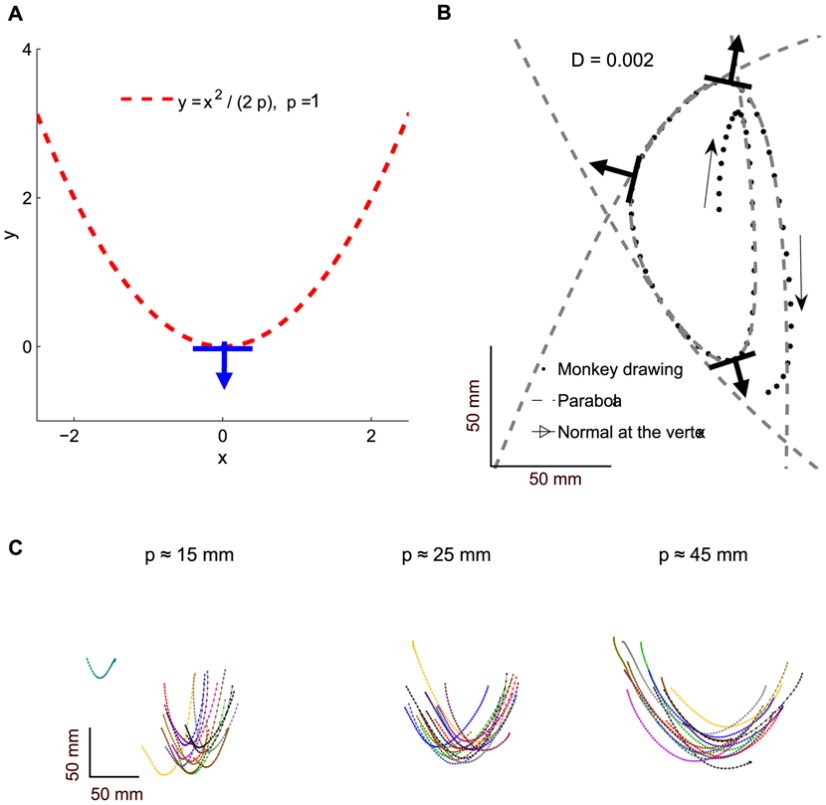
A parabola in the canonical coordinate system and fitted parabolic strokes. A. Parabola is shown using the canonical coordinate system. The orientation of the normal at the point of maximal curvature is 270° and the focal parameter *p* = 1. B. An example of a pattern of monkey drawing that emerged after several practice sessions and could be well approximated by three parabolic pieces with different orientations. C. These parabolic strokes were fitted to monkey drawings. Different strokes have similar orientations and are grouped according to their focal parameter 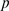.

A parabola is defined by 4 parameters: the focal parameter 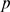, two coordinates of the location of the vertex (point of maximal curvature), and the orientation of the parabola (defined by the direction of the normal vector at the vertex). Direct verification shows that the focal parameter *p* equals the radius of curvature (reciprocal of the curvature) at the vertex of a parabola. Every parabola can be transformed by rigid rotation and translation into the canonical coordinate system in which the orientation of a parabola is 270°, and its vertex is located at the point whose coordinates are *(0, 0)*. In the canonical coordinate system, the parabola is described by a simple relationship with a single free parameter 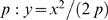. Such a parabola is shown in Figure 3A. The three typical parabolas emerging from the fitting of the monkey drawing (dots) are given in Figure 3B. The mean of their R^2^-based estimate of the goodness of fit 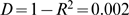 indicates a very good fit. Several typical examples of the fitted parabolas and estimates of the goodness of fit can be found in [27]. The focal parameters, orientations and locations of the vertices of these three fitted parabolas are all different. Note that for rest-to-rest movements through a single via-point, minimum-jerk trajectories can be very well approximated by parabolic segments, though they are not exact parabolas [27]. Such trajectories are characteristic of obstacle-avoidance human movements or curved movements through a single via-point [3].

The fitting of parabolic segments to the monkey hand trajectories was implemented in a consistent way using the greedy algorithm described in Text S2. By consistent, we mean that the outcome of the fit is invariant under those equi-affine transformations of the path which preserve the location of the point of maximal curvature on the fitted parabolic segment. Thus, our procedure applied in the same manner to both “narrow” and “wide” path segments. Examples of “narrow” and “wide” parabolic segments are shown in Figure 3C.

To quantitatively assess the amount of incongruence between any path segment and the corresponding parabolic segment fitted to it, a measure of *discrepancy* was defined. For each segment *i* of the recorded movements, this discrepancy measure was evaluated by calculating the value of 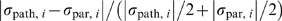, where 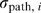 and 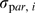 are the estimated equi-affine lengths of that path segment and of the corresponding fitted parabolic segment, respectively. The discrepancy measures ranged from 0 to 2.0. The more similar the two equi-affine lengths are, the smaller is the discrepancy between the recorded path and the parabolic segment fitted to it. Higher discrepancy measures usually correspond to practically straight movement segments which contain inflection points causing larger errors in the numerical estimation of the equi-affine invariants. Equi-affine analysis is not appropriate near inflection points. Examples of movement segments and fitting parabolas corresponding to different discrepancy measures, having low (0.08) to high (1.5) values, are depicted in Figure S1.

## Results

We analyzed the scribbling trajectories recorded during 17 consecutive recording sessions for monkey O and 16 consecutive recording sessions for monkey U. These recording sessions started from the beginning of practice for both monkeys. We also analyzed movement data from 17 recording sessions of *well-trained* behavior in monkey U that were recorded almost a year after the beginning of monkey U's practice. Well-trained data for monkey O were not available.

**Figure 4:**
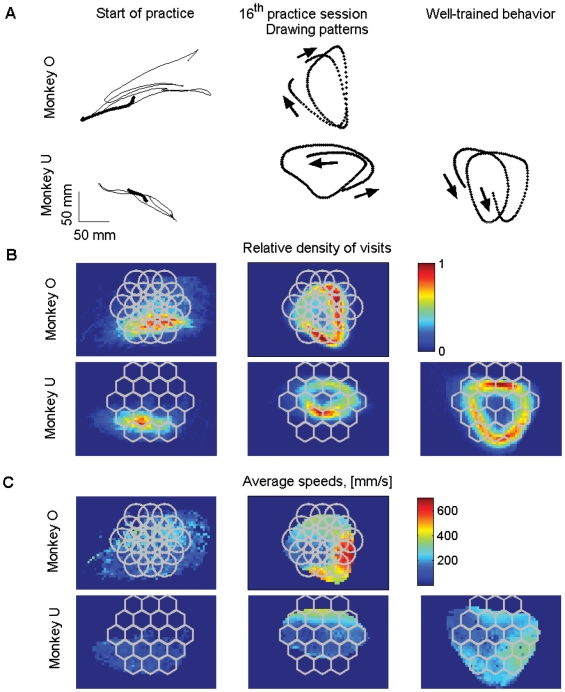
Demonstration of behavior at different stages of practice. A. Paths drawn by monkeys O and U at the beginning of the practice period (left), during the 16^th^ practice session (middle), and path drawn by monkey U during the period of well-trained performance. The dotted segments in each plot have the same duration of 1.98 sec. Although slow and jerky in the beginning, with practice the movements became smoother, faster and more regular. B. Dwell distributions for the end-point position, same sessions as in A. Depicted are the frequencies of visits weighted by tangential velocity; that is, visiting the same location once with a tangential velocity equal to 450 mm/s has the same contribution as visiting 3 times with a tangential velocity equal to 150 mm/s. These weighted frequencies indicate that monkey movements become more stereotypical after a period of practice. C. Average tangential velocities of the end-point, same sessions as in A.

The monkeys began by producing trajectories composed of short and nearly straight movements and with practice converged towards systematically clockwise (monkey O) or counter-clockwise (Monkey U) patterns of movement. The movements became smoother, increasingly simpler and composed of a series of parabolic segments (Figure 4A).

The dwell distributions for the endpoint of the manipulandum (defined in Methods) indicated that the monkey movements became more stereotypical following a period of practice (Figure 4B). The monkey movements became mostly confined to the plausibly rewarded area (target areas). Hence, in our analysis we did not make a distinction between movements within or outside these areas. Figure 4C shows examples of averaged tangential velocity profiles with respect to the locations of the monkeys' hands (speeds are indexed according to the locations of the end-effector and are averaged for each location).

The data analysis produced similar results for both monkeys in spite of the following differences in their behavior:

1. After the first few practice sessions monkey O and monkey U mostly scribbled in opposite directions (clockwise and counter clockwise, respectively), though both monkeys used their right hands.
2. Monkey O scribbled noticeably faster than monkey U.

We started from the direct test of the convergence to piece-wise parabolic behavior by analyzing the properties of parabolas fitted to the scribbling movements. Next we tested the same phenomenon using our definition of a movement primitive. Minimum-jerk modeling and equi-affine analysis of trajectories are described in Text S1.

### Dimensionality reduction and convergence to sequences composed of parabolic-like segments

**Figure 5:**
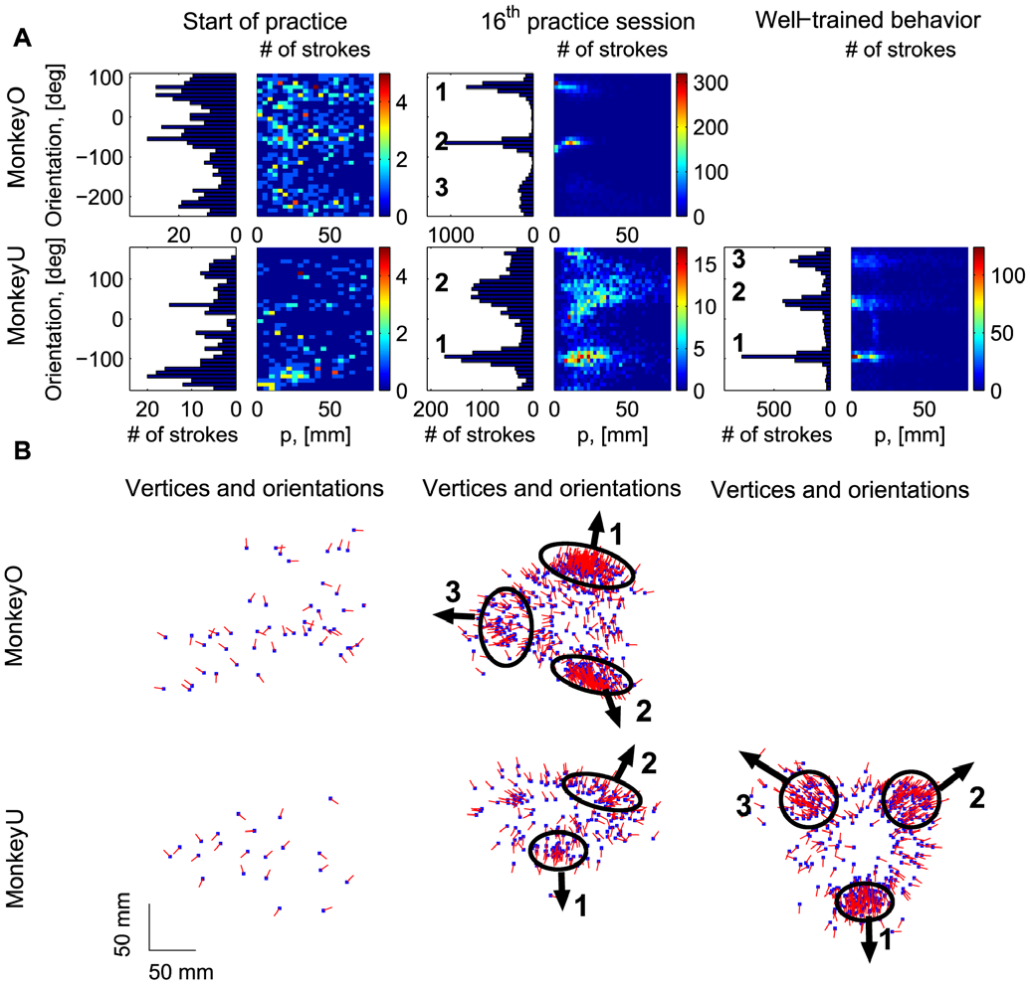
Emerging parabolic clusters and dimensionality reduction. A. Typical histograms for the fitted parabolic segments. In the one-dimensional histogram (left), the segments were tabulated according to their orientation. In the color histogram, they were tabulated in bins identified by two values: the orientation and the focal parameter of the parabola. B. Location of the vertex and orientation of the parabola for every 10^th^ parabolic segment for the recording sessions in (A). Locations of the vertices of the similarly oriented parabolas are also clustered. The clusters are marked by ellipses and the mean orientations of the parabolas within each cluster are indicated by arrows.

After both monkeys had practiced the drawing task, the parabolic segments that were fitted to the recorded movements fell into 2–4 clusters based on their orientation. The focal parameter *p* and orientation 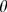 define a unique parabola up to translation (see Figure 3). Figure 5A shows typical histograms of the number of parabolic strokes tabulated according to the values of the quantized pairs of (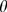, *p*) and according to the orientation parameter 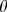. In comparison to the lack of distinct clusters in the histograms for the parabolic segments derived from the beginning of practice, the practiced movements clearly showed convergence to well separated clusters, based on the orientation of the fitted parabolic strokes.

Apart from the parabolas' focal parameter and orientation, we also examined the remaining two parameters that define a parabola, namely the 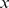 and 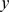 coordinates of the location of the vertex. Figure 5B shows the locations of the vertices of the fitted parabolic segments and their orientations for every tenth fitted parabola (to make the data easier to visualize) from the same recording sessions as in Figure 5A. The example shows that after a period of practice, the locations of vertices of similarly oriented parabolas were separable into distinct clusters as well. In Figure 5, the clusters are labeled 1–3, corresponding to the order of the performed trajectories. Note that monkeys O and U scribbled in opposite directions and therefore the orders of the clusters for the two monkeys are opposite.

**Figure 6:**
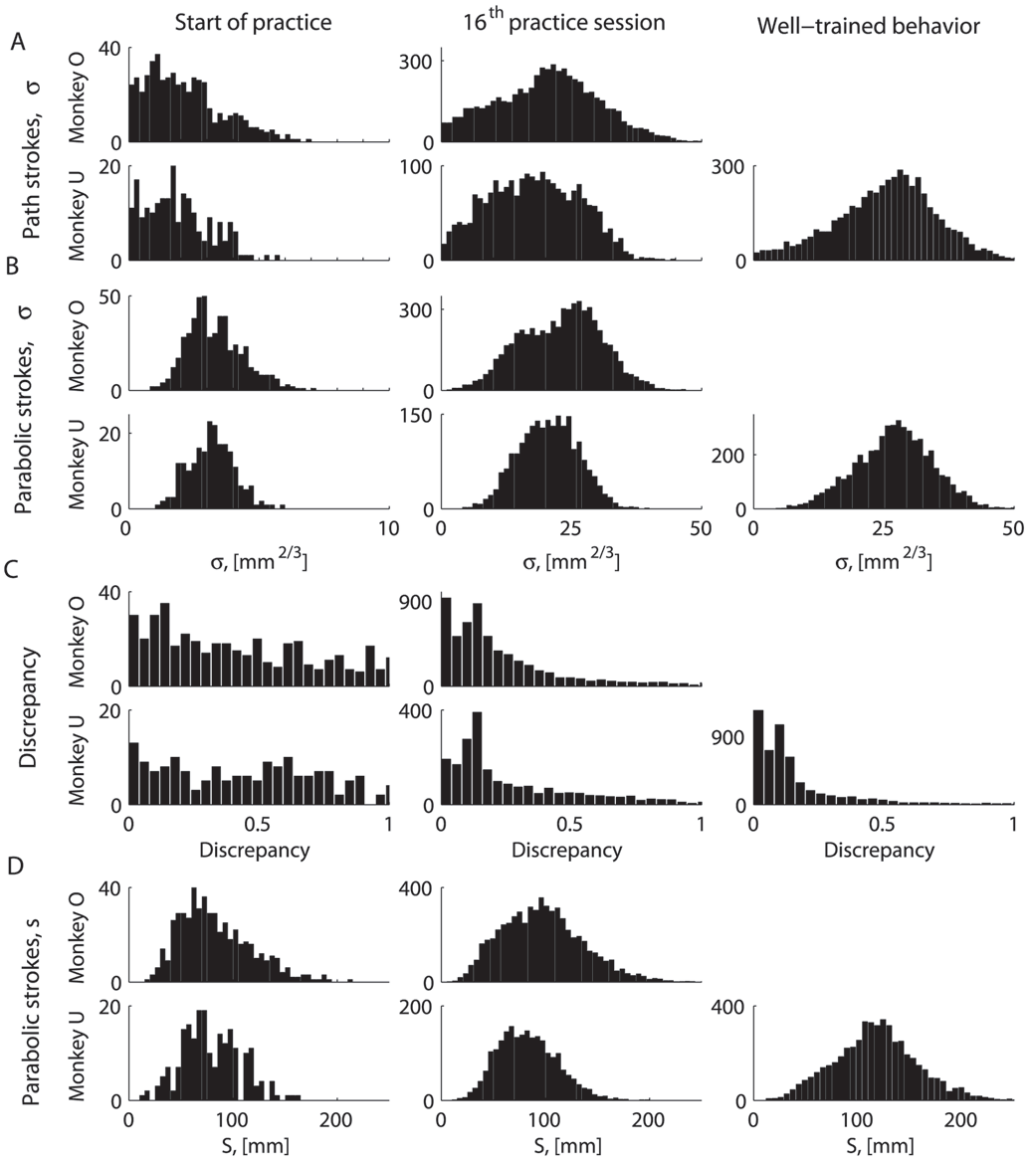
Properties of the paths and fitted parabolic strokes at different stages of practice, demonstration. Histograms of the parameters of the fitted parabolic strokes for recording sessions taken from different practice periods of both monkeys. A. Equi-affine lengths of the path strokes fitted with parabolic strokes. B. Equi-affine lengths of the fitted parabolic strokes. Practice makes the measures from A and B more similar. C. Discrepancy between the path strokes and the parabolic strokes fitted to them. Practice decreases the discrepancy. D. Euclidian lengths of the fitted parabolic strokes.

**Figure 7:**
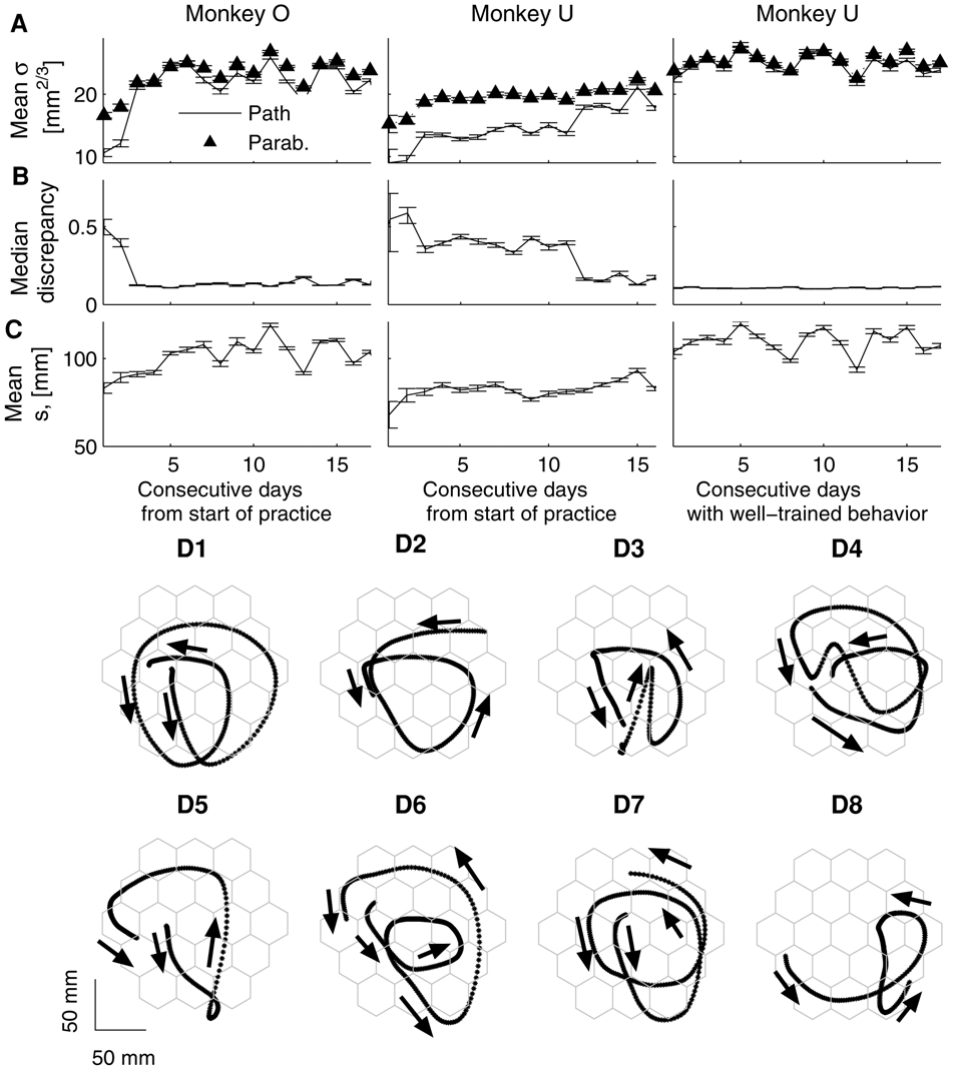
Properties of the paths and fitted parabolic strokes during practice and examples of drawing patterns. A–C. First column: monkey O from the beginning of practice. Second column: monkey U from the beginning of practice. Third column: well-trained behavior of monkey U. A. Mean values of the equi-affine length of the path strokes fitted with parabolas (continuous line), mean values of the equi-affine length of the fitted parabolic strokes (triangles). B. Parabolic discrepancy, an estimate of the deviation of the fitted paths from piece-wise parabolicity. C. Mean values of the Euclidian length of the path strokes fitted with parabolas. D. Different drawing patterns corresponding to the well-trained behavior of monkey U. D1, D2. Correspond to typical patterns described by ordered sequences of parabolic strokes taken from the different identified clusters. The examples depicted in D3–D8 are relatively rare. D3–D5. Different patterns with reversals in movement direction. D6–D8. Movement patterns with “irregular” parabolic strokes, i.e., some of the fitted parabolic strokes fell outside the directionally identified clusters.

Figure 6 shows typical histograms of the equi-affine and Euclidian lengths of the recorded movement strokes and the corresponding parabolic strokes. Histograms of the equi-affine lengths of the monkey path strokes for all different practice periods are depicted in Figure 6A. Histograms of the equi-affine lengths of the parabolic strokes fitted to these path segments are shown in Figure 6B. Corresponding histograms in Figure 6A and 6B are more similar to each other for the sessions that followed a period of practice. This indicates that with practice the equi-affine lengths of the path strokes became more similar to those of the corresponding fitted parabolic strokes (the similarity was assessed quantitatively using the discrepancy measure introduced in Methods). Typical distributions of the calculated discrepancy measures derived for different practice periods are depicted in Figure 6C. Indeed, practice led to a decrease in the values of the discrepancy measures. Euclidian lengths of the fitted parabolic strokes were all quite similar to the Euclidian lengths of the recorded parabolic-like paths (which can be fit well with parabolas) and therefore these lengths are not shown separately. Typical distributions of Euclidian lengths of the fitted parabolic strokes are depicted in Figure 6D. The data depicted in Figure 6 are summarized in Figure 7A–C for all analyzed recording sessions.

Figure 7A shows the mean equi-affine arc lengths of the fitted parabolic segments and the parabolic-like path segments they fit for all the movement recording sessions together with 95% confidence interval. Figure 7B depicts the median values of the discrepancy measures for all the recording sessions analyzed and the corresponding 95% confidence intervals. The median values of the discrepancy measures decreased and the equi-affine lengths of the fitted parabolic segments increased (i.e. the fitted parabolas became longer) as both monkeys had more practice. Euclidian lengths of the fitted parabolic strokes and the corresponding path segments were very similar; they became larger with practice. The average values of the Euclidian lengths for all recording sessions and their 95% confidence intervals are shown in Figure 7C.

The error in fitting parabolic strokes to movement paths was also estimated using the R^2^ measure as described in Text S2. For every recording session analyzed, the median error was very small and in the range of 10^−3^–10^−2^ of data variance. Taking parabolic strokes from all recording sessions together, the resulting value of (1−R^2^) was 2.66×10^−3^ (median; 95% confidence interval: [2.65 2.67]×10^−3^). However an R^2^ based measure, unlike the discrepancy measure, is not sensitive to modifications of the drawn trajectories during practice.

The goodness of fit of the monkeys' drawing movements to other (non-parabolic) curves was also evaluated. In particular, ellipses, higher order polynomials (of orders 3–5) of the form 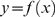 and triplets of superimposed point-to-point movements (which fit parabolic paths quite well, see Text S3) were fitted to the same movement parts that were fitted with parabolas. The R^2^ based measures of the deviation from the recorded paths for all the above-mentioned kinds of curves were small (1−R^2^<0.01). To estimate the trade-off between goodness of fit and model simplicity (number of parameters), the SIC score [35] was used (see [27] for details of using the SIC score). This analysis indicated that out of all the different curves considered here, the parabolic model yielded the highest SIC score. That is, the parabolic model provided the best trade-off. The same conclusion was drawn in [27] for segmentation of the trajectories into parabolic segments using a different segmentation algorithm than the one used in the present manuscript. More specifically, in [27] curves were fitted to movement parts, where the end-points of the fitted curves were anchored at consecutive points of minima of the curve's Euclidian curvature. This fitting scheme did not aim at providing the longest possible path segments which can be well fitted with parabolic strokes by contrast to the fitting scheme used here.

So far we have shown that parabolic segments grouped into clusters over the course of practice and therefore could capture the geometric regularities of the well-practiced movements. We also observed that about 60%–80% of trajectory durations recorded during each session could be well approximated by large parabolic-like segments. Hence, between rest periods and for movements generated within different parts of the workspace the monkeys completed motion sequences which were composed of several piece-wise parabolic segments. Furthermore, when the monkeys became well-practiced, they rarely reversed their movement direction which was either clockwise (for monkey O) or counterclockwise (for monkey U).

The fitted parabolic segments can be labeled according to the clusters to which they belong. Note that the order of clusters for each monkey is identified according to the direction of the drawing; i.e., sequence (1-2-3) for monkey O would correspond to a clock-wise direction of motion, while for monkey U the same sequence of labels (1-2-3) would correspond to a counter-clockwise direction of motion. The clusters corresponding to different monkeys cannot be identically labeled because the monkeys generated movements in different directions. Therefore, as Figure 5 demonstrates, the clusters for monkey O are labeled differently than the clusters for monkey U.

The alphabet of labels for practiced sessions of monkey O consisted of 3 labels because 3 parabolic clusters were found in that session (Figure 5). The sequence (1-2-3) represents a repeatable word because the orientation of the well-practiced drawings was mostly constant. Over the course of practice, the monkeys' drawings could be represented more and more precisely in terms of repeatable sequences of labels identifying parabolic clusters, where each series of labels constitutes a “word”. For example, the drawing of monkey O depicted in Figure 3B can be represented by the sequence (1-2-3)-1, where, following the labeling shown in Figure 5, 1 denotes upward oriented parabolas, 2 denotes downward oriented parabolas, and 3 denotes leftward oriented parabolas. Note that in Figure 3B, the parabola fitting the initial part of the drawing (which corresponds to cluster 1) was not depicted (to make the amount of information depicted in this plot comprehensible). Three parabolas fitted to the scribbling of monkey O (in Figure 3B) are associated with cluster sequence 2-3-1.

In Figure 7D1 and 7D2 we show typical examples of drawing patterns consisting of ordered sequences of parabolic-like strokes. These are quite characteristic patterns including relatively rare cases in which the direction of motion was reversed (Figure 7D3–D5). Nevertheless, in a few cases, elemental parabolic strokes identified as belonging to cluster 1 were not followed by other parabolic elements. This happened either when the movement was stopped or when reversing movement direction. Examples of paths partially composed of the fitted parabolic segments not belonging to any one of the three clusters are depicted in Figure 7D6–D8.

### Analysis of drawing movements based on the two-thirds power law and the constrained minimum-jerk models

Parabolas are the only equi-affine invariant curves which provide identical predictions to the constrained minimum-jerk model and the two-thirds power law [25],[27]. We therefore estimated the degree of fit of the monkeys' scribbling movements to these two models and the detailed description of this analysis is presented in Text S1. In particular, an example of a movement segment, its corresponding equi-affine invariants and the predictions of the two models are presented in Figure S3 (while the procedure of regularizing the equi-affine speed is demonstrated in Figure S2). As Figure S4A and S4B show, monkey scribbling movements deviate to some extent from both models.

Note that the degree of fit of the movements to the predictions of the two models was estimated for movement segments composed of several concatenated parabolic strokes, while the predictions of both models are identical only for a single parabolic segment, and not for sequences of parabolas. Drawing each separate parabolic segment within a sequence at a constant equi-affine speed would lead to very high values of jerk at the transitions between adjacent segments, resulting in non-smooth movements. This implies that although on the geometric level the movements were indeed shown to be approximately composed of simply concatenated parabolas, on a kinematic level constant equi-affine speed could not be maintained. Hence the spatial (geometric) aspects might be planned separately or even precede the temporal aspects of planning (e.g. concatenation is observed only on the geometric level).

Interestingly, the trajectories predicted by the constrained minimum-jerk model fit the two-thirds power law better for more practiced movement paths (see Text S1 and Figure S4C and S4D).

We also examined another kinematic optimality criterion, the minimum-acceleration model, according to which movements tend to minimize an integrated second derivative of the drawn trajectories (rather than the third derivative as in case of the minimum-jerk model). Using the same approach as in the case of the minimum-jerk model in [27], we derived an equation whose solutions define paths providing identical predictions for the two-thirds power law and the minimum-acceleration model:
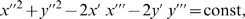
which is equivalent to 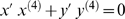 for smooth enough curves. Here a prime denotes differentiation with respect to 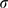, and the numbers in brackets denote the corresponding higher order derivatives with respect to 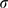. Using the same approach as in [27], it can be shown that parabolas constitute the only class of equi-affine invariant curves satisfying above equation. Nevertheless, there is no ambiguity as to which model provides a better fit for the data. An implementation of the minimum-acceleration modeling to the scribbling paths showed that the degree of deviation of the minimum-acceleration trajectories from the recorded movements was higher than that of the minimum-jerk trajectories.

### Decision strategies, movement variability, and definition of a movement primitive

In this study of the compositional nature of movements, we attempt to go beyond analyzing separate movement components. In particular, we investigate the nature of the underlying movement primitives by examining modifications in scribbling strategies that were associated with well-identified behavioral events: receiving or not receiving a reward. The quantitative analysis was based on using the parabolic components of the recorded trajectories introduced above.

**Figure 8:**
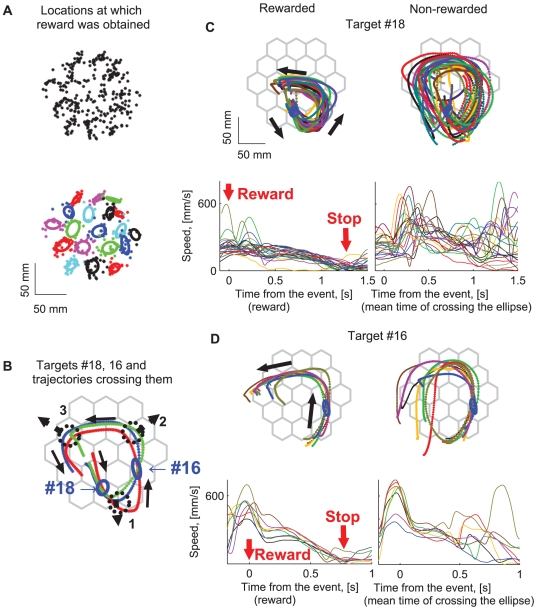
Rewarded and non-rewarded trajectories. A. Selection of areas with high density of rewarded locations. (Upper) Locations at which the monkey received a reward. (Lower) Same locations as in the upper plot, different colors correspond to different targets. PCA ellipses designate areas with a high density of rewarded locations. B. Three movement patterns that cross ellipses #18 and #16 without being rewarded there. For 2 trajectories, red and purple, the monkey completes the primitive sequence (1–2) and continues to scribble further via parabolic element 3 without stopping. For the green trajectory, the monkey completes the primitive sequence as well and decelerates after its completion. If the monkey is rewarded at target 18, it completes parabolic segments 1 and 2 and then nearly stops (see Figure 8C). If the monkey is not rewarded at target 18, and gets a reward at target 16, it completes parabolic segment 2, decelerates and nearly stops after that (see Figure 8D). In general, after obtaining a reward at targets 18 and 16 (at the beginning of parabolic strokes 1 or 2) the monkey decelerates and nearly stops after completing the sequence (1–2) (the data from all 17 recording sessions are summarized in Figure 9A). C, D. After receiving a reward inside ellipses 16, 18 during an ongoing movement the monkey tended to decelerate and even stop moving but only after completing a movement sequence composed of several parabolic segments. The set of rewarded locations is marked by a blue ellipse. The monkey scribbled counter clockwise. C. An example of the completed (before stopping) particular cycle that consisted of two parabolic segments (oriented downward and rightward). D. The last parabolic element of the cycle mostly corresponded to the parabolic strokes whose orientations ranged from 0° to 100°. The trajectories which were not rewarded inside the ellipse were more variable than the rewarded ones, which may have resulted from the composition of an ongoing movement sequence with another movement element.

The effect of rewards on the drawings was especially pronounced in the well-practiced movements of monkey U. After almost a year of practice, monkey U tended to decelerate and sometimes almost stop its arm movement after it had been rewarded. Rewards were obtained near the target boundary (Figure 8A, upper plot). Hence, the locations rewarded within a session did not cover the workspace uniformly. To examine the kinematic differences between rewarded versus non-rewarded trajectories for the movements performed by the well-trained monkey U, in each session, 19 areas within the workspace where the reward density was high were selected based on the 19 targets where the reward was delivered.

For convenience, every such area with a relatively high density of rewarded locations was represented by an ellipse whose main axes correspond to the principal components of the 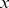 and 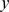 coordinates of the rewarded locations within this area (Figure 8A, lower plot). The lengths of ellipses' main axes are equal to the unbiased standard deviation along the corresponding directions: 
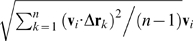
. Here, for 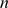 rewarded locations corresponding to target 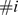, 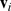 denotes a unit vector parallel to one of the two main axes of the ellipse, and 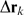 corresponds to the vector connecting the ellipse's center with the 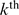 rewarded location identified with this target. In the lower plot of Figure 8A, different colors were used to depict rewarded locations identified with the different targets as well as the corresponding ellipses.

The scribbling strategy that the monkey tended to use consisted of initiating movements within the proximal part of the workspace with respect to the monkey and only then exploring the distal part of the workspace. The rewarded trajectories which passed through the areas of the designated ellipses were compared to movements that crossed these ellipses without being rewarded there. In general, both the rewarded and the non-rewarded trajectories were composed of several piece-wise parabolic segments. A careful analysis indicated clear differences between the rewarded versus the non-rewarded trajectories.

Three examples of unrewarded trajectories are shown in Figure 8B. These movement patterns cross the ellipses centered on targets #18 and #16 without being rewarded there. In 2 of these 3 trajectories, marked in red and purple, the monkey completed the sequence which was composed of primitives (1–2) and continued to further generate parabolic segment 3 without stopping the movement after completing the sequence (1–2). In the case of the trajectory marked in green, the monkey decelerated the movement after completing the sequence (1–2).

Plots in Figure 8C display the hand paths (left panel, upper row) and tangential velocity profiles (left panel, lower row) of the rewarded trajectories from one recording session, passing through the ellipse corresponding to 18^th^ target. Figure 8C also shows the paths and tangential velocity profiles of the non-rewarded trajectories (right panel), from the same recording session as in the left panel, passing through target #18. Similar plots are shown in the corresponding panels of Figure 8D for both the rewarded and non-rewarded trajectories passing through the ellipse corresponding to the 16^th^ target. In the case of the unrewarded trajectories passing through the 18^th^ target, the monkey completed a movement sequence composed of two parabolas (labeled 1–2) and continued the sequence by also completing the third parabolic element. By contrast, for the rewarded trajectories, after being rewarded at target #18, the monkey completed parabolic segments 1 and 2 and then nearly stopped its movement (see Figure 8C). The paths and tangential velocity profiles are clearly more variable for the non-rewarded trajectories.

When rewarded at target #16 and not at target #18, the monkey completed parabolic segment 2, and only then the movement was decelerated and subsequently was nearly halted (see Figure 8D). Hence, in general, after obtaining a reward at targets #18 or #16 (at the beginning of either strokes 1 or 2, respectively) the monkey tended to decelerate and nearly stop its movement only after completing sequence (1–2) in the case of target #18 or element 2 in the case of target #16.

There were other targets which were followed by rewarded movement sequences composed of two parabolas. These targets corresponded to the stage following the initiation of drawing parabolas belonging to cluster 1 (as depicted in Figure 8B). In particular, some of the sequences generated following reward delivery at target #14 consisted of two parabolic strokes. A more general observation follows from inspecting the lower-right plot depicted in Figure 4B which shows a typical path produced by the highly trained monkey U: a parabola belonging to cluster 1 is typically initiated at targets #13, #14 or #17. It typically crosses target #18 only after its initiation, that after crossing either of the targets #13, #14, #17. Therefore trajectories following reward delivery occurring at targets #13, #14 or #17 were typically more variable than those generated following reward delivery at target #18.

For the movements passing through the ellipse centered on target #16 (Figure 8D), similarly to the case of target #18, both rewarded paths and tangential velocity profiles were more stereotypical than non-rewarded paths and tangential velocity profiles. When plotted time is aligned on the event of receiving a reward at target #16, or the mean time of crossing the boundary of the ellipse of target 16 for the non-rewarded trajectories, the rewarded trajectories showed a clear halt within about 0.8 sec following the reward with no such clear halt for the non-rewarded trajectories.

Hence, based on these observations, we operationally define a movement primitive as a movement entity that cannot be intentionally stopped before its completion once it has been initiated. Furthermore, the above observations indicate the existence of “words” or “sentences” composed of several parabolic-like strokes (e.g. sequences (1–2)) which serve as higher level geometric primitives.

Following the observations of the influence of drawing strategies on movement variability described above, for all 17 recording sessions with a well-trained behavior (Monkey U), we then quantitatively examined the validity of the claim that the monkey indeed tended to slow down and almost stop movement after receiving a reward at targets #18 or #16 but only after either being able to complete the drawing of a parabolic element (in the case of target 16), or after completing the generation of a sequence composed of 2 parabolic segments (in the case of target 18). Since the monkey could complete a sequence of 2 parabolic-like segments within 1–2 s., (see the left panel in the lower part of Figure 8C) it was assumed that the monkey nearly stopped its movement within a time interval of 1 to 2 s after receiving a reward as compared to simply passing through target ellipse #18 without being rewarded there. Similarly, for target #16 we assumed that the monkey nearly stopped its movement within a time interval of 0.5 to 1 s from the time it was rewarded (see the left lower panel of Figure 8D).

**Figure 9:**
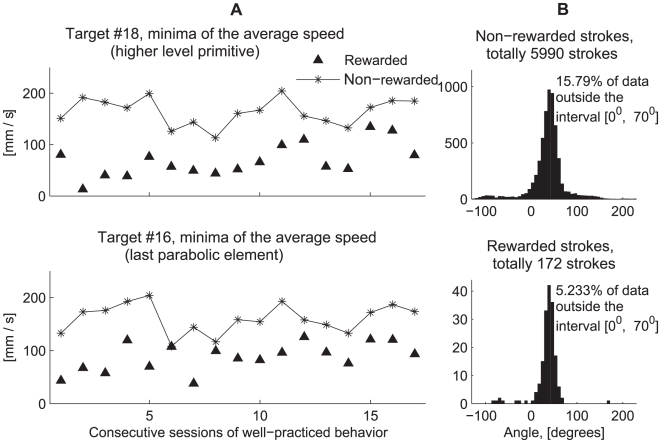
Differences between the rewarded and non-rewarded trajectories, statistical estimates. Differences between rewarded and non-rewarded trajectories. A. Minimal values of the average tangential velocities of the rewarded and non-rewarded trajectories for the sequence (1–2) which is a higher level primitive (target #18, as in Figure 8C) and for the last parabolic element (target #16, as in Figure 8D). B. Corresponds to parabolic strokes fitted to the trajectories constituting the last parabolic element (ellipses corresponding to target #16, as in Figure 8D). The orientations of 15.8% of the non-rewarded and 5.23% of the rewarded segments were outside the orientation interval [0, 70] degrees. This difference in variability was statistically significant (see text).

The speeds (corresponding to the above-mentioned time intervals) across rewarded and non-rewarded trajectories were further averaged. The graphs in Figure 9A show that for trajectories rewarded inside ellipses #16, #18, the average values of the hand speeds at their minima were always smaller than the velocity threshold which we used to mark periods of rest (i.e. 150 mm/sec, Methods), thus indicating that the movements were nearly halted after receiving a reward. They were also always smaller than the minima of the average speed for the corresponding non-rewarded trajectories. The differences between the minimal tangential velocity values of the rewarded versus the non-rewarded trajectories averaged across all 17 sessions were significant (Mann–Whitney U test, p = 0.05).

The ellipse corresponding to target #16 (see Figure 8B and 8D) was specifically chosen because it is positioned at the location which corresponded to the beginning of the parabolas constituting the last elements in the sequence (1–2). This allowed us to use our parabolic fitting algorithm to estimate the degree to which a single movement primitive isolated from other movement elements was indeed stereotypical. For 10 of the 17 recording sessions (1–5, 10, 14–17), we visually observed a greater variability in the non-rewarded trajectories that crossed ellipse #16 versus the trajectories that were rewarded inside this ellipse. We then defined a time interval of 0.05–0.4 seconds from the event (of either getting a reward or the mean time of crossing the ellipse boundaries in case the reward was not obtained). As can be seen in the lower left plot of Figure 8D, this interval includes two local maxima and one local minima of the tangential velocity of the trajectories crossing the ellipse. This tangential velocity pattern usually corresponded to a single parabolic-like drawing.

For each trajectory examined, we selected the point with the highest path curvature for the time interval of 0.05–0.4 seconds from the event. A parabola containing this point was then selected. All the rewarded trajectories shown in Figure 8D could be fitted with parabolas oriented at about 50° as compared to more variable orientations of the parabolas fitted to the non-rewarded trajectories. These parabolic elements from the rewarded and non-rewarded trajectories were then used to statistically demonstrate the greater variability of the trajectories that crossed the ellipse #16 without being rewarded there as compared to trajectories rewarded inside this ellipse.

Figure 9B demonstrates that the orientations of the rewarded segments are concentrated within the interval [0°, 70°]. In fact, orientations of only 5.2% of the rewarded segments lay outside this interval, compared to 15.8% of the non-rewarded segments. This implies that parabolic strokes identified with the non-rewarded trajectories which crossed the ellipse corresponding to target #16 (Figure 8D) were more variable than the parabolic strokes fitted to the trajectories rewarded inside this ellipse (binomial test, p = 0.01). These parabolic elements of the rewarded trajectories may be less variable because the movement nearly stopped and was not followed by a consecutive movement element (whose choice could be based on making a decision).

The upper left plots in Figure 8C and 8D show that the locations of the termination of the parabolic stroke belonging to the 2^nd^ cluster mainly correspond to the second quadrant of the workspace (locations above and to the left of the central target); i.e., these locations are not uniformly distributed within the workspace. In order to quantitatively analyze this phenomenon, we define the location at which the movement stopped as the end of a movement segment having a speed threshold of 100 mm/s within 2 seconds after an event occurred. Such an event involved either getting a reward or corresponded to the mean time of crossing the ellipse boundary when the reward was not obtained. In cases when the end of a movement segment did not occur within 2 seconds after such an event, the location of a halt to movement was defined as the location at which the speed was the lowest within a 2 second interval after the event. Further analysis of the movements (both rewarded and not rewarded) which crossed all targets 1–19, and not only targets 16 and 18, showed that indeed the locations of movement halts were not uniformly distributed within the workspace.

Taking all targets together, 40.36% of the rewarded trajectories stopped (as defined in the paragraph above) within the second quadrant of the workspace whereas only 29.8% of the non-rewarded trajectories stopped within this quadrant. The difference was significant (binomial test, p = 0.01). Therefore, frequent stopping within the second quadrant of the rewarded trajectories was related to getting a reward and not simply to the monkey's purported intention to stop there irrespective of the preceding movement history. Rather, stopping within the second quadrant of the workspace supports the notion that a post-rewarded sequence was halted after completion of the last element belonging to the 2^nd^ cluster more often than a non-rewarded sequence.

In some cases, although the monkey tended to decelerate its motion after receiving a reward, it did not entirely stop the movement. Because of the existence of such cases, there were post-rewarded movement sequences with more than 2 parabolic elements. However, the number of post-rewarded sequences rapidly decreased with the increase of the number of their elements and the sequences composed of 1 or 2 post-rewarded parabolic elements constituted 52.2% of all the post-rewarded sequences. Only one post-rewarded sequence contained 20 elements and this sequence was the longest one observed for all the recording sessions in the well-trained monkey U. Post-rewarded sequences with more than 2 elements mainly corresponded to the sessions with a higher speed of motion; e.g. sessions 15 and 16 as seen in the upper plot of Figure 9A.

## Discussion

We analyzed modifications in spontaneous scribbling movements of two monkeys over the course of practice and the properties of well-practiced scribbling movements. Our results show that after the monkeys practiced with the motor task, the variability of the parabolic pieces fitted to these movements, decreased and could be classified as belonging to only 2–4 groups of parabolas, clustered according to their orientations. The lengths of the fitted parabolic strokes also tended to increase. This finding therefore indicates that a reduction in the dimensionality of the generated movements evolved through practice. This reduction in dimensionality indicates that parabolas capture essential features of the fitted drawings, a property that is required from any possible movement primitive in order to be considered as a likely building block in movement compositionality. Analysis of motor cortical activity recorded during the same scribbling movements also suggests that the emergence of parabolic patterns has central origin [27].

By analyzing differences between the rewarded and non-rewarded trajectories, we introduced a general definition of a movement primitive defined at the geometric level of hand trajectory planning. This analysis also suggests that decision-making processes should be taken into account in studies of movement compositionality.

### Parabolic primitives

Fitting parabolas to the scribbling movements robustly allowed us to determine the focal parameters and orientations of the fitted parabolic strokes. We also fitted parabolas to the paths which connect consecutive local minima of the Euclidian curvature. The orientations of these parabolic segments formed clusters which were similar to those derived based on the orientations of the parabolic strokes obtained through the application of the greedy algorithm applied here (see Methods). Note that we fitted parabolas to the movement data and did not decompose the trajectories into the underlying strokes. That is, each shape was fitted independently of the others and an arbitrary amount of overlap between two consecutive fitted shapes was allowed.

An important implication of fitting parabolas to the recorded movements is the dimensionality reduction of the data. In our fitting procedure, each parabola corresponds to a single local maximum of Euclidian curvature. No two maxima of Euclidian curvature correspond to the same parabola and no two parabolas correspond to the same maximum; that is, there is no ambiguity among movement elements. The existence of overlaps between consecutive parabolic elements allows for smooth transitions between these elements and strengthens our claim that the movements are well described by parabolic segments.

However, there were also gaps between consecutive parabolic elements. The gaps corresponded either to occasional very slow motion within segments or to nearly straight motions. The case of occasional very slow motion within movement segments does not correspond to actively preplanned movements and therefore is not relevant to our analysis [27]. The case of nearly straight movements (occurring near inflection points) cannot be treated within the framework of equi-affine geometry because the equi-affine length of straight paths is zero and their equi-affine curvature is not defined. Straight movements are geodesics in Euclidian geometry and should be treated within the framework of Euclidian geometry, while parabolas are equi-affine geodesics [24],[36].

Our study suggests the existence of a central representation of movements in terms of parabolic primitives. The emergence of the recorded parabolic-like patterns during practice cannot be described solely as a reflection of the generation of smoother movements per se [27]. Considering the fit with non-parabolic curves (ellipses, polynomials of order 3–5, and triplets of superimposed point-to-point movements), although the fit is very good and superior to the fit with parabolas, parabolas provide the best trade-off between goodness of fit and simplicity of the curve and are equi-affine invariant. As regards parsimony of representation, a complicated planar curve can be represented by means of an affine invariant model composed of parabolic polygons [37]. Hence, parabolic strokes cannot be considered as simply useful basis functions selected only because they provide a successful numerical approximation to the recorded movements.

Our data were limited to the end-effector locations and therefore our analysis did not address the issue of what degree of motion smoothness combined with the biomechanical properties of the limb may have led to the observed piece-wise parabolic movement paths. Concerning the origin of the smoothness of hand trajectories, several empirical and modeling studies have proposed that muscle properties by themselves are sufficient to account for much of the observed smoothness and bell-shaped speed profiles which are characteristic of point-to-point movements, e.g. [38]–[40]. It has also been argued that the two-thirds power law also originates from low pass biomechanical properties of the muscles, e.g. [41], or other peripheral factors such as the effects of the non-linearities of the forward kinematic transformations [42]–[44], or the inherent noise present in the motor system [45].

Nevertheless, a number of studies have supported the central origin of the two-thirds power law. These include, for example, the demonstration that variations in the magnitudes and directions of the neural population vectors are consistent with the kinematic properties of monkey trajectories that obey the two-thirds power law [46],[47]. Our analysis also showed that the firing rates of some of the cells in the motor cortical area (recorded while the monkeys were performing the drawing movements reported here) were more strongly correlated with the equi-affine movement speed rather than with Euclidian speed [25],[27]. The two-thirds power law also affects the perception of motion. Movements performed according to the two-thirds power law are perceived as being more uniform [20],[21]. Recently [26], demonstrated that compliance with the two-thirds power law in motion perception is reflected in stronger fMRI activations of different cortical regions and in particular, of brain areas that subserve motor production, visual motion processing, and action observation functions. Interestingly, analysis of equi-affine invariant primitives in planar movements has been recently generalized to the spatial case in empirical and theoretical studies [25], [27], [48]–[50].

Earlier studies proposed the existence of movement primitives at different hierarchical levels, suggesting corresponding syntactical rules for combining them in order to compose complex movements; for more details see [51]. The use of geometric primitives in our study differs from other investigations, as we suggest a specific malleable geometric shape acted upon by a group of geometric transformations, without a vectorial basis of units of composition. This differs from vectorial superposition of elements from a fixed set of basic functions/force fields/muscle synergies, e.g. [11]–[13],[52],[53].

Our results suggest that parabolic movements identified in monkey scribblings may, through the process of practice, form a behavioral output of dynamically-switching cortical “attractors” (states) [27]. Convergence toward attractor-like neural activity during practice via Hebbian learning may underlie the superposition/co-articulation of sequences of point-to-point motion units into more compact and smoother parabola-like movement components. Rehearsal of a sequence of elementary planar point-to-point movements by human subjects leads to the formation of more complex smooth geometric primitives [31], thus supporting this suggestion. Moreover, the smooth movements that emerged following practice were well approximated by minimum-jerk trajectories passing through a single via-point. Geometrically, such minimum-jerk trajectories have a parabolic-like shape; therefore the geometric primitives observed by [31] might be parabolic-like segments.

A single parabolic drawing can be approximated with three superimposed point-to-point movements, each having a bell-shaped speed profile (Figure S5A). Each bell-shaped speed profile can, in turn, be approximated with three smaller identical bell-shaped speed profiles, and so on (Figure S5B). However, a triplet of point-to-point movements is defined by at least seven parameters while a parabola is defined by only four parameters, thus providing a more compact representation. One possibility is that in the hierarchy of geometric primitives, point-to-point movements constitute the lowest level which is below the hierarchy of curved movements; another possibility is that there is no hierarchical relationship between straight and curved movement primitives (see also Text S3). Elementary parabolic-like shapes (“letters” of the “alphabet” used to achieve a compact representation) constitute the lowest level in the hierarchy of curved movements, and sequences of parabolic-like shapes (“words”) belong to the next level above. Elementary parabolic primitives and their sequences are acquired during learning to achieve more efficient representation of complex well-trained movements (in terms of complexity) as the present study has demonstrated.

The observed reduction in variability of the fitted parabolic components of the scribbling movements indicates a tendency of the CNS to increase the parsimony of movement representation through practice (hypothesis of greater parsimony). The geometric reduction of dimensionality in drawing movements also has an equi-affine interpretation. Piece-wise parabolic trajectories can be generated based on affine transformations (equi-affine transformations and spatial scaling) of a single parabolic template; equi-affine curvature of monkey scribbling movements became closer to zero through practice [27],[30]. Parabolas have a constant zero equi-affine curvature. Therefore, empirical evidence for greater parsimony implicitly imposes a geometric constraint on the movement path in terms of the equi-affine curvature. This role of the equi-affine curvature, in turn, suggests that equi-affine variables and geometry may play an important role in the representations employed by the primate motor system.

Our demonstration of the dimensionality reduction that was achieved through practice also enabled us to represent spontaneous movements in terms of sequences of elementary primitives and finally to introduce a compact symbolic notation to describe the recorded (continuous) scribbling data based on a small (discrete) set of basic primitives. Moreover, mathematically, piece-wise parabolic movements can be viewed in this perspective as resulting from applying sequences of different affine geometric transformations to a single movement template (parabolic), rather than constituting different movement elements. Hence, the experimental paradigm and the movement analysis approach described here may serve future studies that focus on human and primate acquisition of motor sequences (see [54],[55] for reviews of such studies).

### Equi-affine invariants, kinematic optimality and geometric properties of drawing movements

Earlier works studied the predictions of the minimum-jerk model for a number of geometric paths and compared corresponding predicted and recorded trajectories [4],[19],[56]. Here, the fit to the constrained minimum-jerk model and the two-thirds power law was estimated for monkey trajectories recorded at different stages of practice.

Geometrically identified patterns were acquired by the monkey through practice but no (or only a slight) influence of practice on the fit to the constrained minimum-jerk model and to the geometric constraint formalized by the two-thirds power law model was detected (Text S1). This may follow from an underlying dissociation between the geometric and temporal aspects of motion planning. The existence of such dissociation was also proposed in earlier studies [57]–[59].

Human tracing movements [19] were found to fit the constrained minimum-jerk model better than monkey scribbling movements [30]. This difference may be due to task differences: the monkeys performed spontaneous scribbling movements, while the human subjects were instructed to repetitively follow prescribed geometric templates.

For non-straight paths, zero jerk cost can be achieved only by drawing a single parabolic stroke and the corresponding minimum jerk trajectory also satisfies the two-thirds power law [27],[30]. However, the constrained minimum-jerk model and the two-thirds power law are not simultaneously satisfied when considering a sequence of parabolic segments. Even so, for monkey scribbling movements, the trajectories predicted by the constrained minimum-jerk model for the recorded monkey paths obeyed the two-thirds power law better after a period of practice (Text S1). The variability (deviation from being constant) of the equi-affine speed was compared for the actual and predicted trajectories to estimate how well a geometric constraint is satisfied by the trajectories predicted by the minimum-jerk criterion versus the recorded trajectories. Future studies of motor learning may similarly apply the comparison of geometric invariants (e.g. equi-affine speed) for actual and modeled trajectories to detect acquisition of specific motor strategies (in our case, modifications of the geometric properties of the movements being performed (Text S1)).

It has been suggested that the motor control signals used in movement generation are chosen such that the end-point variance should be minimized [6]. It was shown in [6] that the trajectories predicted by the minimum end-point variance for the drawing of ellipses fit the two-thirds power law well. Thus, the equi-affine speed of these predicted motions is close to being constant. Note as well that when drawing an ellipse according to the constrained minimum-jerk model, the predicted trajectories also fit the two-thirds power law quite well [56], and that parabolas can be approximated arbitrarily well by ellipses with close to zero equi-affine curvatures [27]. Therefore, the predictions of the minimum-variance model for a parabolic path would yield trajectory which fits well the two-thirds power law, similarly to the case of the minimum-jerk model.

### Compositionality of movements, movement variability and decision-making

Our observations, based on the comparison between the kinematic characteristics of the rewarded versus the non-rewarded trajectories have shown that receiving or not receiving a reward affected the motion sequences generated by a well-practiced monkey. Significantly smaller variability was observed for the parabolic strokes fitted to the trajectories rewarded at a specific spatial location (corresponding to the start of the last element in the sequence) versus parabolic strokes fitted to the non-rewarded trajectories. The last elements of the rewarded trajectories may be less variable due to the fact that when the movement is nearly halted, it is not followed by a consecutive movement element which is composed with the preceding one. Therefore, the observed differences in movement variability between the rewarded versus the non-rewarded trajectories lend support to our definition of a movement primitive. Our observations also indicated that parabolic segments constitute elementary motion primitives which are used in the construction of higher-level sequences, i.e., “words” or “sentences” in well practiced scribbling movements. Consequently, we propose that the observed behavior of the well-trained monkey could imply that the monkey has applied a strategy of combining a few parabolic parameters into higher-level sequences.

However, it is not entirely clear why the monkey tended to concatenate the parabolic elements belonging to clusters 1 and 2 into an indivisible sequence. That is, why didn't the monkey immediately halt its movement when it received a reward at target #18, but tended, instead, to continue generating another parabolic element and then arresting the motion after its subsequent completion? All trajectories which were rewarded at either targets #16 or 18 were stopped only after competing element 2 in the sequence. This finding, in turn, may imply that the monkey employed a movement generation strategy which involved automatic exploration of the distal part of the workspace (parabolic cluster 2) in case no reward was obtained within its proximal part (parabolic cluster 1).

The findings reported here indicate that complex movements may be generated by tuning the parameters of a small number of primitives and then concatenating them together to achieve the goals of complex movements. For example, a likely parameter to be tuned is the focal parameter of the parabolic-like segments which defines their “width”. Tuning of primitives in goal-directed movements may also be guided by decision-making and/or action selection based on ongoing feedback/reinforcement signals (e.g. receiving or not receiving a reward). Therefore, paradigms involving decision-making could be advantageous in studies investigating movement construction based on the compositionality of a basic repertoire of motion primitives. In fact, a recent study involving the analysis of rapid pronation/supination wrist movements produced by monkeys during a 1D step-tracking task indicated that a decision-making process guided the initiation of corrective sub-movements [60].

Our preliminary observations also indicated that some of the motor cortical units may be responsive to receiving a reward and thus their activity may be related to decision-making processes [25]. Our monkey data were recorded in a paradigm in which the monkey did not have a clear motivation to stop its movements after reward delivery. However [61], studied the performance of human subjects when generating free scribbling movements in which the subjects were looking for the location of an invisible target and were requested to unexpectedly impede their movements. Geometrical analysis of the recorded trajectories showed that the figural properties of the paths generated after the “stop” cue was given were part of a repetitive geometrical pattern and that the probability of completing a pattern after the “stop” cue was given was correlated with the relative advance in the geometrical plan rather than with the amount of time that had elapsed since the initiation. Thus the findings of [61] provide evidence in support of the existence of movement primitives which subserve the construction of sentence-like sequences in human trajectory formation. Their observations therefore support our claim that a primitive can indeed be defined as an entity that cannot be stopped before its completion.

Is the convergence of monkey drawings to trajectory sequences composed of several parabolic-like segments an optimal strategy in terms of a sequential search for rewards? Does it reflect the development of a geometric skill based on core knowledge [62], or is it the outcome of the development of a dynamic internal model, or are both inherently related? Further studies involving, for example, non-uniform or even non-stationary distributions of target locations and studies using dynamic perturbations of arm movements (in both humans and monkeys) should provide further answers to these questions. Such studies could also examine the possible existence of parabolic primitives and the degree of involvement of decision-making mechanisms in movement compositionality and variability. We also propose that the relative simplicity of movement data (versus acoustic or semantic data, for example) makes their analysis a useful tool in studies dealing with problems of binding and cognitive processing.

In summary, different kinematical analysis and mathematical modeling approaches were combined in our study and indicated that with practice, monkey scribbling movements tend to be composed of parabolic elements drawn from a small number of directionally identified clusters. The observed piece-wise parabolicity of the movement segments is also compatible with our general definition of movement primitives and the notion that repeated practice of a given motor task leads to a more parsimonious motor representation.

## Supporting Information

Text S1 Application of the equi-affine geometry and minimum-jerk modeling to analysis of movement kinematics.
(0.10 MB PDF)

Text S2 Procedure for fitting parabolas.
(0.03 MB PDF)

Text S3 Approximation of parabolic-like trajectories with triplets of point-to-point minimum-jerk movements.
(0.01 MB PDF)

Figure S1 Discrepancy measure. Examples of movement segments and fitting parabolas corresponding to different discrepancy measures, from low (0.08) to high (1.5). Higher discrepancy usually corresponds to close to straight movement parts which contain inflection points.
(0.02 MB PDF)

Figure S2 Illustration of the regularization procedure. Left: Given a sequence of values of |Δσ|, some of its elements are not close enough (as defined in the text) to their neighbors; e.g. the first element is not close to the second. We assume that a regular piece of data consists of at least 5 consecutive elements that are close enough to each other. Right: The regularized sequence of the parameter.
(0.02 MB PDF)

Figure S3 An example of kinematic and equi-affine analysis for a movement segment. A. Segment path. B. A dashed-dotted graph shows the time evolution along the segment had the monkey drawn the path in A according to the constrained minimum-jerk model. C. Drawing speeds. The x axis corresponds to time for the profiles (a) and (b), and to the sample point number divided by the recording frequency, which is identical to time for actual trajectories, for profiles (a), (c). Minima and maxima of the actual and predicted trajectories occur at similar positions on the path (comparison of (a) and (c)), but their time-course is different (comparison of (b) and (c)). D. Scaled magnitudes of the regularized (with outliers omitted, see Text S1) increments of the equi-affine arc-length, scaled increments of the predicted time intervals between adjacent samples and equi-affine curvature. Several segments with an equi-affine curvature close to zero can be seen. E. Equi-affine speeds, actual and predicted (superscripts a and p respectively), were scaled to fit the same axes as in D. The predicted equi-affine speed deviates from being constant less than the actual equi-affine speed as measure γ from the formula (S8) in Text S1 indicates.
(0.06 MB PDF)

Figure S4 Fit to the constrained minimum-jerk model and to the two-thirds power law. A. Averages of the estimated fit of the trajectories to the constrained minimum-jerk model. For both monkeys the estimates lay within the same range. No convergence can be seen. B, C, D. Averaged estimates of the non-constancy of the actual and predicted equi-affine velocities. B. Fit of the actual trajectories to movement segments according to the two-thirds power law. On average, the fit of the trajectories of monkey O did not change through practice. Monkey U showed some improvement of the fit. C. Both monkeys showed a clear improvement with practice in the fit of the predicted trajectories to the two-thirds power law compared to the beginning of practice. D. For both monkeys, the fit of the predicted trajectories to the two-thirds power law was better than the fit of the actual trajectories. The superiority in fit of the predicted trajectories increased through practice, especially for monkey O.
(0.05 MB PDF)

Figure S5 Approximation of a parabolic-like trajectory with three point-to-point movements. A (upper part). A parabolic-like path and three point-to-point movements. The approximation is marked by dashed lines. Although this result is demonstrated for a single parabolic-like trajectory, its affine transformations can be applied in case of other parabolic segments (to reconstruct parabolic-like path). A (lower part). Speed profiles of the parabolic-like trajectory (blue), approximating trajectory (dashed) and point-to-point movements. All speed profiles correspond to the respective paths from the plot above. B. Point-to-point minimum-jerk trajectory can be composed of 3 identical minimum-jerk trajectories rescaled in time and space. The ratio of peak speeds is approximately 0.55.
(0.03 MB PDF)
