# Supplementary material for: A Compact Representation of Drawing Movements with Sequences of Parabolic Primitives
Source: PLoS Comput Biol. 2009 Jul 3;5(7):e1000427. doi: 10.1371/journal.pcbi.1000427 (PMC2699652; doi:10.1371/journal.pcbi.1000427)
Supplement: Text S1 — Application of the equi-affine geometry and minimum-jerk modeling to analysis of movement kinematics. (0.10 MB PDF) [file pcbi.1000427.s001.pdf]

Supporting Text 1

Application of the equi-affine geometry and minimum-jerk modeling to analysis of movement kinematics

Here we describe the procedure for numerical modeling of the trajectories satisfying the constrained minimum-jerk model and compare the modeled movements with the recorded data. We also use equi-affine speed to estimate the fit of the measured and predicted movements to the two-thirds power law model. We found that the temporal course of the monkeys' movements deviated from the prediction of the constrained minimum-jerk model; however the model successfully captured the locations on the path at which the tangential velocity achieved extremal values. The fit of the predicted trajectories to the two-thirds power law improved with the amount of practice the monkey had, which is not the case for the actual recorded trajectories.

The notions of equi-affine geometry and the rationale for its application in motor control studies are described elsewhere [22-25, 27, 32, 33]. Here we provide essential definitions, explanations, and methods of analysis of movements' kinematic parameters. An English translation of the relevant material from [32] can be found in [25] (Appendix A).

Essential notions of the planar equi-affine differential geometry

Planar affine transformations,

$$\begin{pmatrix} x^* \\ y^* \end{pmatrix} = A \cdot \begin{pmatrix} x \\ y \end{pmatrix} + \begin{pmatrix} a \\ b \end{pmatrix}, \text{ where } A = \begin{pmatrix} \alpha & \beta \\ \gamma & \delta \end{pmatrix},$$

which are constrained by the condition $\det A = 1$, are called equi-affine transformations. These include a linear part defined by 3 independent terms appearing in the matrix A; where the fourth term depends on the other 3, provided that $\det A = 1$. It also includes translation components a and b. The variables x and y are coordinates of a point located along a planar curve, and x^* and y^* are the coordinates of the same point following the equi-affine transformation. The condition on the determinant means that the area contained within any closed curve is preserved under equi-affine transformations; these transformations are therefore also called area-preserving. The set of Euclidian transformations consists of rigid rotations and translations that preserve Euclidian distance and curvature, and constitutes a particular subset of equi-affine transformations. An equi-affine transformation can be applied to all the points along a curve. The Euclidian length of a curve and its Euclidian curvature are modified accordingly. However the equi-affine arc-length and the equi-affine curvature, defined below, remain the same.

For a twice differentiable planar trajectory described by the vector function $\mathbf{r}(t) = \{x(t), y(t)\}$, the equi-affine velocity, which is equal to the time derivative of the equi-affine arc-length σ, is invariant under equi-affine transformations and is expressed as follows [32, 33]:

$$\dot{\sigma}(t) = \sqrt[3]{\dot{x}(t)\ddot{y}(t) - \dot{y}(t)\ddot{x}(t)}\,. \qquad \text{(S1)}$$

Here and elsewhere, a dot above a symbol denotes a time derivative and boldfaced symbols signify vector quantities.

It equals the cubic root of the area of the parallelogram defined by the vectors of the movement velocity and acceleration and is invariant under equi-affine (area-preserving)

transformations. Similarly, the well-known formula $\dot{s}=\sqrt{\dot{x}^2+\dot{y}^2}$ for the Euclidian speed of motion is invariant under the action of the group of Euclidian transformations (rotations and translations). Equi-affine length σ can be computed either by integrating equi-affine velocity (S1) over time or based on Euclidian invariants as the integrated Euclidian length s weighted by the cubic root of the Euclidian curvature c:

$$\sigma(t)=\int_0^{L(t)} c^{1/3}(s)ds\,,$$

where L is the Euclidian length of the trajectory at time t.

For a trajectory $\mathbf{r}(t)$ having no inflection points (where the Euclidian curvature is zero), and parameterized by the equi-affine arc length σ (called natural parameterization), a derivative w.r.t. σ is denoted by a prime $\left(x'=dx(t(\sigma))/d\sigma=\dot{x}/\dot{\sigma}\right)$, where $t(\sigma)$ corresponds to the movement duration during which an equi-affine distance σ (mm$^{2/3}$) of the path is drawn. Given that for an equi-affine transformation $\det A=1$ (see equation 1 above), the condition for an equi-affine parameterization is:

$$x'y''-y'x''=1. \tag{S2}$$

Equation (S2) corresponds to the area spanned by the equi-affine tangent $(\mathbf{r}')$ and by the equi-affine normal $(\mathbf{r}'')$ to the curve. Note that the expression in equation (S2) is equal to the outer product and not to the scalar product of the two vectors. Differentiation of equation (S2) with respect to σ shows that the vectors corresponding to the first and third derivatives of the position vectors are parallel to each other and thus can always be related to each other by some scalar κ:

$$\mathbf{r}'''+\kappa\mathbf{r}'=0\,, \tag{S3}$$

with the proportionality coefficient κ obtained from equations (S2) and (S3) being expressed as

$$\kappa(\sigma)=x''y'''-y''x''' .$$

The coefficient κ is the equi-affine curvature of a curve [32, 33], which is another equi-affine invariant. Curves having the same equi-affine curvature can be aligned by using some equi-affine transformation [32]; therefore equi-affine curvature can be used for curve classification in equi-affine geometry.

Curves having a constant equi-affine curvature are the conics (the ellipse, parabola, and hyperbola). Parabolas have zero equi-affine curvature. Therefore any parabolic stroke can be uniquely associated with any other parabolic stroke, whenever the two strokes have the same equi-affine length [27, 30]. For this reason any parabolic segment can be obtained from an arbitrary parabolic template by an affine transformation (composed of a unique uniform spatial scaling – adjusting the equi-affine length – and a unique equi-affine transformation). The equi-affine curvature of an ellipse is a positive constant defined only by its enclosed area A: $\kappa=(\pi/A)^{2/3}$ [34].

The constrained minimum-jerk model predicts maximally smooth movements in the jerk sense for a given movement path [4]; more details are provided in the next section. Predictions of the constrained minimum-jerk model and the two-thirds power law model for a given path are generally different. They are equal only for curves satisfying the following equation [25, 27]:

$$x'''^2+y'''^2-2x''x^{(4)}-2y''y^{(4)}+2x'x^{(5)}+2y'y^{(5)}=\text{const} , \qquad \text{(S4)}$$

which is equivalent to $x'x^{(6)}+y'y^{(6)}=0$ for smooth enough curves. Here the prime denotes differentiation with respect to σ, and numbers in brackets denote the

corresponding order of differentiation with respect to σ. Parabolas constitute the only class of equi-affinely invariant solutions of this equation [25, 27].

Actual and predicted trajectories

Let $\mathbf{r}(s)=\{x(s), y(s)\}$ be a planar curve describing the path of the hand during a particular trial, where s is the Euclidean distance along the path and $\dot{s}(t)$ is the hand tangential velocity. The constrained minimum-jerk model [4] assumes that the law of drawing a given geometric path during movement duration T is defined by the scalar function $s(t)$ which minimizes the integrated jerk, namely

$$J=\frac{1}{2}\int_0^T \left\{\dddot{x}^2[s(t)]+\dddot{y}^2[s(t)]\right\}dt \,. \qquad \text{(S5)}$$

The optimization procedure described below was aimed to find a prediction of the constrained minimum-jerk model or, alternatively, a speed profile $\dot{s}(t)$ that minimizes the cost (S5). The optimal trajectory is constrained by a prescribed path $\{x(s), y(s)\}$ and the total duration T of a movement segment. No constraints were imposed on the velocity or acceleration at the segment boundaries as we seek for predictions along movement segments (by definition, the monkeys' hand velocity was always non-zero along the movement segments, see Methods).

A classical minimum-jerk trajectory for moving from rest to rest passing through a single via-point (3 points constrain the task instead of the entire path) can be very well approximated by a parabola, though it is not an exact parabola [25, 27]. The constrained

minimum-jerk model predicts movements with constant equi-affine speed, that is satisfying the two-thirds power-law model, for parabolic paths.

Given a sequence of recorded samples along the path of a movement segment, $\{x_i, y_i\},\ i=1,\ldots,N$, the predicted time intervals of traveling between adjacent samples $\mathbf{\Delta t}^p = \{\Delta t^p{}_1,\ldots,\Delta t^p{}_{N-1}\}$ with $\sum_1^{N-1} \Delta t^p{}_i = T$ must be estimated to define a trajectory minimizing the jerk along that path. Once samples along a movement path and time increments were obtained, the sample-wise components of the jerk cost were calculated by means of the following Matlab code:

```
function J = Jerk (X, Y, Dt)
DtAcceleration = 1/2 * (Dt(1:end-1) + Dt(2:end));
DtJerk = 1/4 * Dt(1:end-2) + 1/2 * Dt(2:end-1) + 1/4 * Dt(3:end);
VelX = diff[X] ./ Dt; VelY = diff(Y) ./ Dt;
AccX = diff(VelX) ./ DtAcceleration; AccY = diff(VelY) ./ DtAcceleration;
jerks_X = diff(AccX) ./ DtJerk; jerks_Y = diff(AccY) ./ DtJerk;
J = 1/sqrt(2) * [jerks_X .* sqrt(DtJerk); jerks_Y .* sqrt(DtJerk)];
```

Finally, the jerk cost (S5) can be approximated with the squared norm of J: `sum(J .* J)`.

The time increments were adjusted during the process of cost minimization implemented with the Matlab (Mathworks) function "lsqnonlin" from the Optimization toolbox. The function was run with the large-scale algorithm provided by the toolbox. The initial guess for the time increments was taken from the recorded trajectory: $\Delta t^a{}_i =$ 1/(Recording frequency); $i=1,\ldots,N-1$.

The predicted time instants $t^p{}_{i+1} = \sum_{k=1}^{i} \Delta t^p{}_k$, $\left(t^p{}_1 = t^a{}_1 = 0\right)$ for moving through the specified recorded positions $\mathbf{r}_{i+1}$, did not precisely match the recorded time instants $t^a{}_{i+1} =$

i / (Recording frequency). A non-trivial time-warping relationship between the actual and predicted trajectories was needed to align the predicted and measured time courses and is defined as follows:

$$w_i = t^p{}_i - t^a{}_i \;,\; i = 1,\ldots,N,\; \left(w_1 = w_N = 0\right). \qquad \text{(S6)}$$

We show below that the predicted trajectories differ from the actual trajectories.

Estimates of the fit to the constrained minimum-jerk model and to the two-thirds power law

For each movement segment, the goodness of fit of the recorded trajectory to the trajectory predicted by the constrained minimum-jerk model was estimated based on time-warping between the two trajectories as follows:

$$\rho_t = \frac{2\cdot 100}{(N-1)T}\left[\sum_{i=1}^{N}|w_i| + \frac{1}{8}\left(|w_2| + |w_{N-1}|\right)\right] \qquad \text{(S7)}$$

with $w_i = t_i^p - t_i^a$ from (S6). The part of this formula in square brackets represents the deviation between the actual and predicted time-courses. The time-course is a strictly increasing function; thus the possible cumulative difference between the actual and predicted time-courses cannot be larger than $(N-1)T/2$, which is set to constitute 100% of the cumulative deviation in the square brackets in (S7).

Now we explain formula (S7) in more detail. Let us use the following notation: $\Delta t = T/(N-1)$, which is equal to the time-interval between consecutive recorded samples of the actual trajectory. The second term in square brackets corresponds to two time intervals $[0, \Delta t/2]$ and $[(N-2+1/2)\Delta T, (N-1)\Delta t]$ of duration $\Delta t/2$ each, whereas the

first term corresponds to the time interval $\left[\Delta t/2,\,(N-2+1/2)\Delta t\right]$ of duration $(T-\Delta t)$. We interpolate linearly between w_1 and w_2 to find the value of w at the point $\Delta t/4$ in the middle of the time interval $\left[0,\Delta t/2\right]$. Given that $w_1 = w_N = 0$, the result of such linear interpolation is equal to $w_2/4$. Numerical integration of this constant value over the interval $\left[0,\Delta t/2\right]$ results in the term $w_2\,\Delta t/8$. The value $w_{N-1}\,\Delta t/8$ is obtained similarly for the time-interval $\left[(N-2+1/2)\Delta t,\,(N-1)\Delta t\right]$. Given that the sequence of predicted time course t_i^p increases with i but never exceeds T and that $w_1 = w_N = 0$, we conclude that the total maximal possible difference between the actual and predicted time-courses (w), integrated over the movement duration is equal to half the area of the squared time interval T: $\left[(N-1)\Delta t\right]^2/2 = (N-1)\Delta t\,T/2$. This maximal possible value is set as 100% and is used in the normalization of the deviation between the time-courses of the recorded and predicted trajectories. The term Δt is cancelled by the same term which is used in the summation (for integration, $\int_0^T |w|\,dt \approx \Delta t \cdot \sum_{i=2}^{N-1} |w_i| + (\Delta t/8)\cdot(|w_2| + |w_{N-1}|)$) in the numerator. Therefore, the normalization factor is $(N-1)T/2$.

The two-thirds power law establishes a relationship between geometric and temporal properties of hand trajectories. It assumes that the tangential velocity $\dot{s}$ for producing a given path and the Euclidian curvature c of that path are related via a piece-wise constant gain factor K: $\dot{s} = K\,c^{-1/3}$ [18]. The gain factor K in the above expression is equal to the equi-affine velocity of the movement trajectory, namely: $\dot{\sigma} = \sqrt[3]{\dot{x}\,\ddot{y} - \ddot{x}\,\dot{y}}\;(=K)$. Therefore, movements obeying the two-thirds power law have

piece-wise constant equi-affine velocity [22-24]; this property is invariant under equi-affine transformations of the trajectory.

The constancy of the ratio $|\mathbf{\Delta\sigma}|/\mathbf{\Delta t} = \{|\Delta\sigma_1|/\Delta t_1, \ldots, |\Delta\sigma_{N-1}|/\Delta t_{N-1}\}$ is used to test whether a given trajectory complies with the two-thirds power law. Whenever the ratio is constant, the angle between the two multidimensional vectors is zero. Therefore, for each movement segment the constancy of the ratio is estimated by the angle between the multidimensional vectors $|\mathbf{\Delta\sigma}|$ and $\mathbf{\Delta t}$:

$\gamma(\mathbf{\Delta t}^a, |\mathbf{\Delta\sigma}|)$ for the actual trajectories*;*

$\gamma(\mathbf{\Delta t}^p, |\mathbf{\Delta\sigma}|)$ for the predicted trajectories *;*

with

$$\gamma(\mathbf{\Delta t}, \mathbf{\Delta\sigma}) = \arccos \frac{(\mathbf{\Delta t} \cdot |\mathbf{\Delta\sigma}|)}{\|\mathbf{\Delta} t\| \cdot \|\mathbf{\Delta\sigma}\|} \quad . \tag{S8}$$

The smaller the angle in (S8)*,* the closer the equi-affine speed is to being constant for a given movement segment and therefore the better the fit to the two-thirds power law.

Regularization of $|\Delta\sigma|$

Numerical calculation of the equi-affine parameters is sensitive to noise in the original data because high-order derivatives are used. Therefore, these parameters occasionally show large fluctuations between adjacent samples. We introduce a regularity criterion for the magnitude of the increments of the equi-affine arc-length $|\Delta\sigma|$ between adjacent samples on a movement path. The criterion is based on the proximity of the neighboring values of $|\Delta\sigma|$. As is illustrated in Figure S2, a block of data is considered

regular whenever it contains a sufficient number (at least 5) of consecutive $|\Delta\sigma|$ that are close enough to their neighbors (0.075 $mm^{2/3}$ or less). The data analysis, which involved equi-affine speed, was performed on those parts of movement segments satisfying the regularity conditions for $|\Delta\sigma|$.

Example: parameters analyzed for a single movement segment

Equi-affine geometry, the two-thirds power law and the constrained minimum-jerk model were used to mathematically infer that parabolas are candidate movement primitives. We next describe the properties of the equi-affine curvature of the monkey scribbling movements and how well these movements fit the two-thirds power law and the constrained minimum-jerk model. First, we use one movement segment scribbled by monkey O to describe in detail the parameters involved in the data analysis presented below.

The segment path (Figure S3A) is smooth and consists of several repetitions of the same piece-wise parabolic pattern. Figure S3B shows the actual and predicted time-courses versus path samples. Their difference defines the time-warping needed for the recorded trajectory to obey the constrained minimum-jerk model.

Figure S3C shows the speed profiles of the actual and predicted trajectories. For the actual trajectories, the sampling interval is proportional to time due to the constant recording frequency. For the predicted trajectories, however, the time taken to pass between the pairs of consecutive samples is not constant. Hence, we plotted a single profile for the actual speed and two profiles for the predicted speed: one was plotted as

a function of time and the other as a function of the sample point number divided by recording frequency (position).

The similarity of speed profiles (a) and (c) in Figure S3C indicates that the constrained minimum-jerk model accurately predicts the locations along the path drawn by the monkeys at which the predicted speed achieves its extremal values. However, the model was less successful in predicting the temporal values of these events, as profiles (a) and (b) indicate. The difference between the actual and predicted time-courses from equation (S6), which defines the time-warping relationship, is exactly the difference between the values along the x-axis at which the graphs (b) and (c) obtain the same values.

Had the predicted trajectory obeyed the two-thirds power law, its velocity gain factor $K^p(t) = \dot{\sigma}^p(t)$ should have been piece-wise constant and, hence, the sequences $\{\Delta t_i^p\} = \mathbf{\Delta t}^p$ and $\{|\Delta\sigma_i|\} = |\mathbf{\Delta\sigma}|$ should have been piece-wise proportional to each other. In Figure S3D, both parameters were scaled to display them on the same axis. The estimated predicted ($|\mathbf{\Delta\sigma}| / \mathbf{\Delta t}^p$) and actual ($|\mathbf{\Delta\sigma}| / \mathbf{\Delta t}^a$) equi-affine speeds were also appropriately scaled and plotted in Figure S3E. The scaling does not change a sequence's deviation from constancy but helps to visualize it. The scaling constants are indicated in the legend of the plot.

As demonstrated in Figure S3D, the scaled sequences of $\mathbf{\Delta t}^p$ and $|\mathbf{\Delta\sigma}|$ follow the same phase of low frequency oscillations and have similar depths of modulation during each phase, disrupted by some higher frequency noise. This indicates that the predicted equi-affine speed $|\dot{\sigma}^p|$ is closer to being constant than the actual one.

The legend in Figure S3E shows the deviation from a constant value estimated by the angle between the multidimensional vectors $|\Delta\boldsymbol{\sigma}|$ and $\Delta\mathbf{t}^p$ or $\Delta\mathbf{t}^a$ for the predicted and actual trajectories respectively, according to formula (S8). The deviation of the actual equi-affine speed from being constant is indeed larger than that of the predicted speed, which demonstrates that the predicted trajectory fits the two-thirds power law better than the actual movement. This is also generally true, as will be shown below, and this is consistent with the fact that an ideal fit to the two-thirds power law is equivalent to setting the normal component of the jerk vector $\dddot{\mathbf{r}}$ to zero [4].

The third graph in Figure S3D demonstrates the values of the equi-affine curvature for a movement segment. Several 'continuous' pieces with equi-affine curvature values very close to zero can be observed in the graph, indicating the possible applicability of the equi-affine curvature to movement segmentation. The values of the equi-affine curvature oscillate around zero, with the local maxima mostly positive and the minima mostly negative.

Fit of the scribbling movements to the constrained minimum-jerk model

The session averages of the deviations between the actual and predicted trajectories estimated by formula (S7) ranged between 4% and 9% (Figure S4A). For both monkeys the averages showed no tendency to converge with practice. For comparison, we estimated the degree to which human tracing movements fit the constrained minimum-jerk model. Human subjects were required to trace 3 specified geometric templates –

double ellipses, cloverleaves and oblate limacons [19]. Average intra-shape deviations of the human tracing movements from the constrained minimum-jerk predictions did not exceed 2% [25] (see Appendix G), which means that they fitted the trajectories predicted by the model better than the monkey scribbling movements. However, the task of the monkeys was to produce spontaneous scribbling, while the human subjects had to follow a predefined geometrical form with a prescribed overall tempo.

Analysis of scribbling movements based on the two-thirds power law and minimum-jerk model

The averages of the estimates of the fit to the two-thirds power law for the actual and predicted time courses and comparisons between them are shown in Figure S4B, C, and D respectively, together with their 95% confidence intervals. Following four recording sessions, the fit of the predicted trajectories to the two-thirds power law became noticeably better, especially for monkey O, and was superior to the fit of the actual trajectories. The degree of fit of the predicted trajectories to the two-thirds power law is dictated by geometric properties of the paths, which serve as the model's constraint. Therefore, improvement of the fit directly implies that the geometric properties of the drawn paths change with practice.

The prediction of the constrained minimum-jerk model for parabolic segments (which has zero jerk cost) satisfies the two-thirds power law [25, 27], which is equivalent to drawing at a constant equi-affine speed. However, this is true for isolated parabolic segments and is not true for their sequences, because such an effect would result in an abrupt change in movement acceleration and thus in high values of jerk. Indeed, the

accelerations of both the actual and predicted movements were not constant. Whether drawing near piece-wise parabolic trajectories (whose paths are sequences of parabolic-like strokes) according to the two-thirds power law is advantageous over other shapes in terms of the jerk cost is a question for further investigation. The transition between adjacent parabolic segments follows a nearly straight path (e.g. as in Figure S3A), i.e., the only path besides a parabola for which the minimum-jerk prediction has minimal possible (zero) jerk cost.
