# Supplementary material for: A Compact Representation of Drawing Movements with Sequences of Parabolic Primitives
Source: PLoS Comput Biol. 2009 Jul 3;5(7):e1000427. doi: 10.1371/journal.pcbi.1000427 (PMC2699652; doi:10.1371/journal.pcbi.1000427)
Supplement: Text S2 — Procedure for fitting parabolas. (0.03 MB PDF) [file pcbi.1000427.s002.pdf]

Supporting Text 2

Procedure for fitting parabolas

We observed that following practice, the fitted parabolic segments converged to a few clusters according to their orientation. Furthermore, the locations of the parabola vertices matched the locations of maximal Euclidian curvature points. This led us to conserve the point of maximal curvature (vertex) in the fitting procedure, which was implemented as follows.

1. Segmentation step: We segmented the data into movement segments (defined in Methods).
2. Initialization step: Points of local maxima of the Euclidian curvature were used as landmarks for fitting the parabolas. For every local maximum of the Euclidian curvature, we selected 9 consecutively recorded samples so that the 5th sample corresponded to the location of the maximal curvature point for that path portion. Note that in this algorithm the Euclidian curvature was estimated for the movement data after they were filtered using a Gaussian filter. This was based on a cut-off frequency of 4 Hz, used for removing multiple small peaks in the estimated curvature, and not on a cut-off of 8 Hz as was used for data smoothing in the rest of our data analysis.
3. Propagation step: We applied a greedy propagation algorithm to these initialized elements of the hand path.

The greedy algorithm for fitting a parabola to the path of a drawing movement is used to iteratively propagate the initialization of the parabolic segment forwards and backwards in time as follows:

1. At each iteration step, find the best fitting parabola for the current path stroke by minimizing the Euclidian distance between the parabola (identified by its focal parameter, orientation and vertex) and the path stroke. Compute the distances between the parabola and 15 position samples backwards and forwards in time along the path stroke.
2. Estimate the number of consecutive samples deviating from the parabola by less than a threshold of 0.4 mm for both the past and future path portions. This threshold value was heuristically selected. If one side contains a larger amount of such samples, propagate the parabolic segment towards that part by one sample; otherwise, if both sides contain an equal (non-zero) number of such samples, propagate towards each side by one sample; otherwise, stop the propagation. Perform all estimations of the deviation in the coordinate system in which the focal parameter of the best fitting parabola is equal to 1 mm. To do so first apply an equi-affine transformation that changes the focal parameter p (to 1) and preserves the location of the point of maximal curvature on the parabola. Incorporating other criteria for deviation from the best fitting parabola, for example, varying the threshold along the parabola may increase the robustness of the algorithm.

An alternative procedure for finding the best fitting parabola for a piece of drawing is to parameterize the path using the equi-affine arc-length σ and fitting it with second order polynomials in σ. This fitting procedure only requires estimating the equi-affine length and does not involve curve fitting at each iteration step. The σ-based fitting procedure is computationally faster and easier to program but demands high precision in

the calculation of the equi-affine length for sample-wise data, which is sometimes difficult to achieve. We propose that finding the best fitting second order polynomial for the path parameterized with the equi-affine arc-length may be useful in further applications which involve fitting parabolas to curves.

We estimated the goodness of fit of the parabola by using the variability of the recorded (x_i, y_i) and parabolic $(x_i, \tilde{y}_i)$ position samples (x coordinates of the recorded and parabolic samples are the same, $\tilde{y}_i = x^2/(2p)$) within the canonical coordinate system for the fitted parabola (defined in step 3 of the greedy algorithm). The error of fitting the recorded stroke was further estimated using the R-square measure as follows:

$$D = 1 - R^2 = \frac{\sum \left(y_i - x_i^{\,2}/(2p)\right)^2}{\sum \left[\left(x_i - \text{mean}(x)\right)^2 + \left(y_i - \text{mean}(y)\right)^2\right]}.$$

We interpret D as the proportion of the data variance unexplained by the model.
