# Supplementary material for: A Compact Representation of Drawing Movements with Sequences of Parabolic Primitives
Source: PLoS Comput Biol. 2009 Jul 3;5(7):e1000427. doi: 10.1371/journal.pcbi.1000427 (PMC2699652; doi:10.1371/journal.pcbi.1000427)
Supplement: Text S3 — Approximation of parabolic-like trajectories with triplets of point-to-point minimum-jerk movements. (0.01 MB PDF) [file pcbi.1000427.s003.pdf]

Supporting Text 3

Approximation of parabolic-like trajectories with triplets of point-to-point minimum-jerk movements

The formation of smooth, parabolic-like drawing shapes may be based on smooth interpolation between two line elements or between required initial and final movement directions as was suggested in [24, 36]. Alternatively, it is also possible that the larger parabolic strokes described here are themselves composed of smaller and simpler straight point-to-point sub-movements based on a vectorial superposition mechanism similar to the one proposed in [11, 52], as shown below.

Minimum-jerk trajectories passing through a single via-point (which fit obstacle-avoidance movements well [3]) are parabolic-like [27]. Such parabolic-like trajectories can actually be constructed using only 3 straight point-to-point movements based on a vectorial superposition mechanism similar to the one proposed in [11, 52], as demonstrated in Figure S5. Near parabolic minimum-jerk trajectory (solid) and the path (dashed) reconstructed by composing 3 point-to-point movements are depicted in the upper part of Figure S5A.

Any parabolic segment can be obtained by an affine transformation of another arbitrary parabolic segment [27]. Therefore, although the result in Figure S5A is demonstrated for a single parabolic-like path, its affine transformations can be applied in case of other parabolic segments (to reconstruct parabolic-like path). The speed profiles of the underlying point-to-point trajectories and the composed trajectory are depicted in the lower part of Figure S5A. Figure S5B shows that any point-to-point movement with a

bell-shaped tangential velocity profile can be well-approximated by 3 identical shorter straight segments that are appropriately scaled in time and space. Altogether, Figure S5 demonstrates how parabolic-like trajectories can be generated hierarchically from arbitrarily short elementary point-to-point movements with bell-shaped speed profiles.

One possibility is that following practice the monkey builds a repertoire of smooth parabolic-like drawing shapes constructed based on the co-articulation of several nearly short and straight sub-movements. In the hierarchy of geometric primitives, point-to-point movements could be considered as corresponding to the lowest level, single parabolic-like shapes ("letters") are one level above, and sequences of parabolic-like shapes ("words") belong to a higher level. Another possibility is that there is no hierarchical relationship between straight and curved movement primitives
