# Supplementary figures and images for: A Compact Representation of Drawing Movements with Sequences of Parabolic Primitives
Source: PLoS Comput Biol. 2009 Jul 3;5(7):e1000427. doi: 10.1371/journal.pcbi.1000427 (PMC2699652; doi:10.1371/journal.pcbi.1000427)

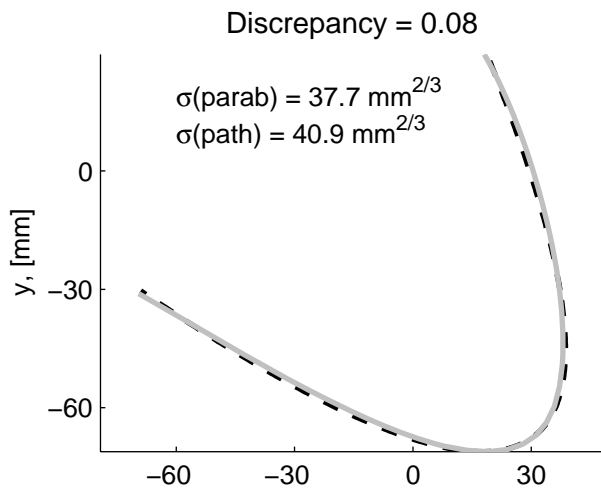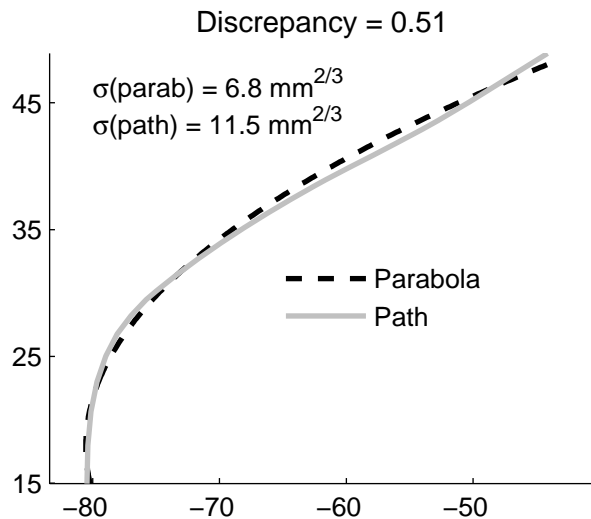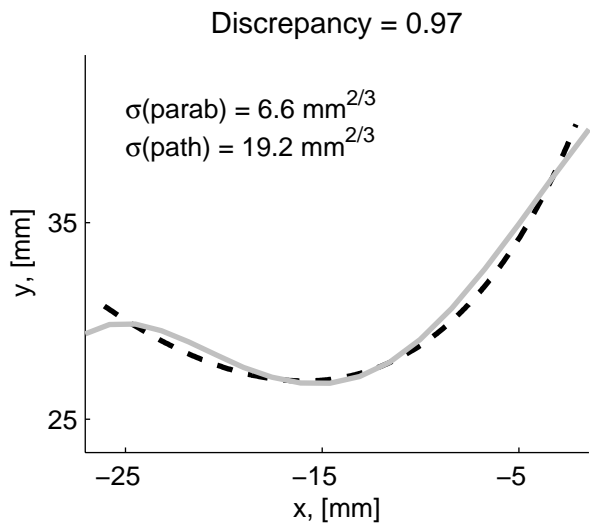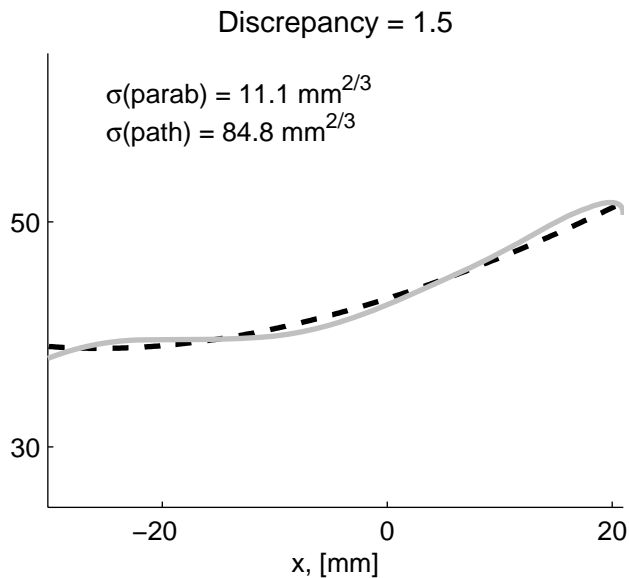

Supplement: Figure S1 — Discrepancy measure. Examples of movement segments and fitting parabolas corresponding to different discrepancy measures, from low (0.08) to high (1.5). Higher discrepancy usually corresponds to close to straight movement parts which contain inflection points. (0.02 MB PDF) [file pcbi.1000427.s004.pdf]

Two regular strokes within a sequence  $|\Delta\sigma|$ 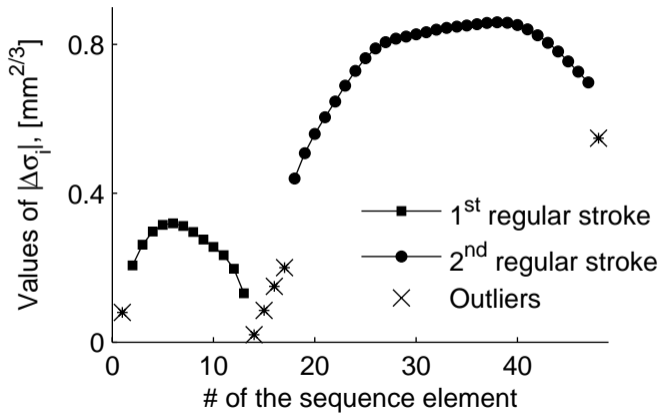

Regularized sequence

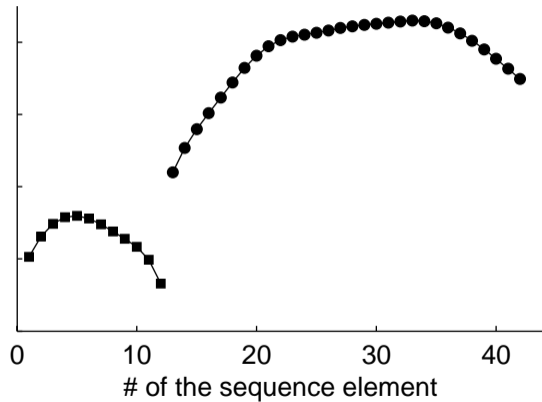

Supplement: Figure S2 — Illustration of the regularization procedure. Left: Given a sequence of values of |Δσ|, some of its elements are not close enough (as defined in the text) to their neighbors; e.g. the first element is not close to the second. We assume that a regular piece of data consists of at least 5 consecutive elements that are close enough to each other. Right: The regularized sequence of the parameter. (0.02 MB PDF) [file pcbi.1000427.s005.pdf]

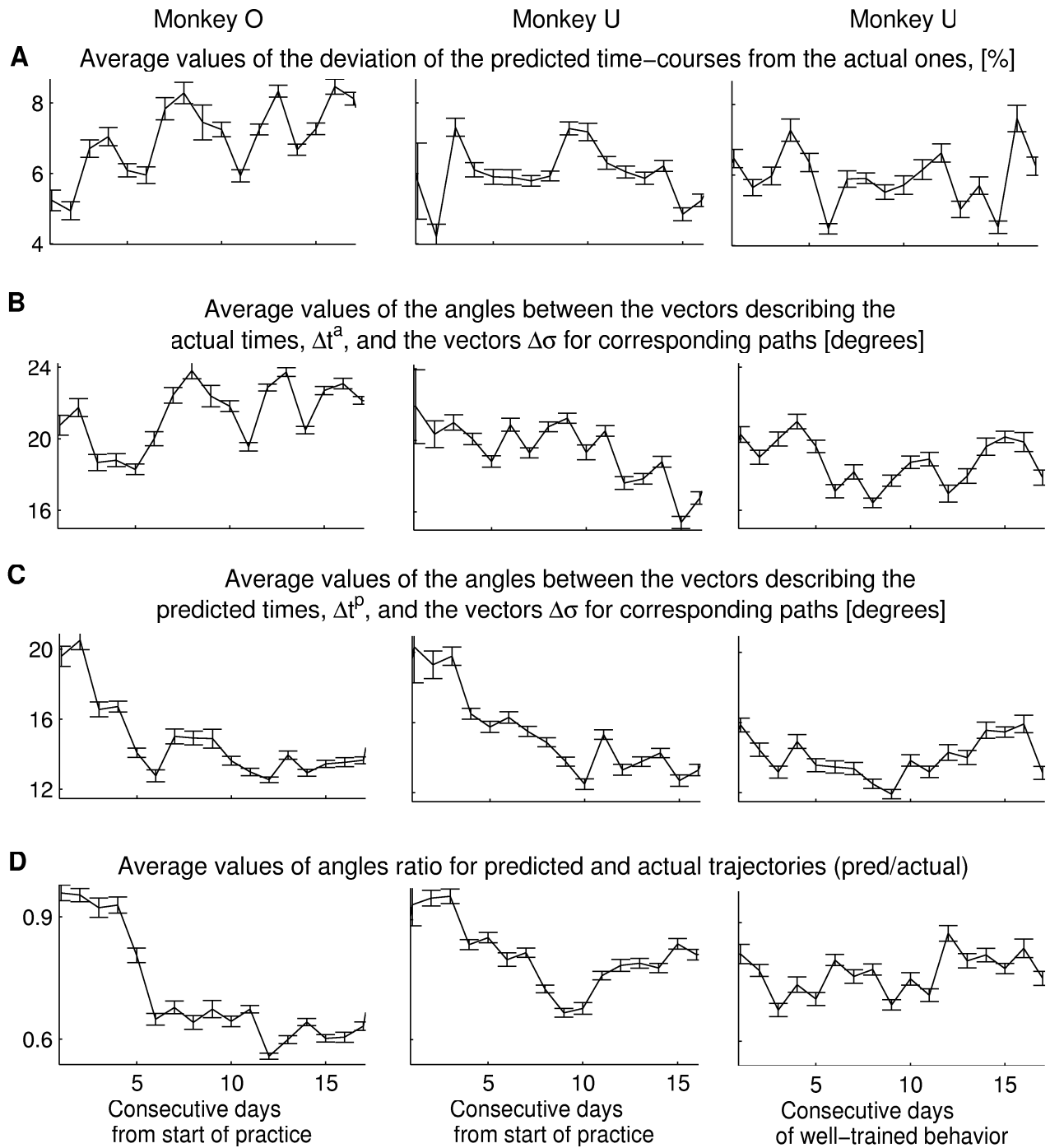

Supplement: Figure S4 — Fit to the constrained minimum-jerk model and to the two-thirds power law. A. Averages of the estimated fit of the trajectories to the constrained minimum-jerk model. For both monkeys the estimates lay within the same range. No convergence can be seen. B, C, D. Averaged estimates of the non-constancy of the actual and predicted equi-affine velocities. B. Fit of the actual trajectories to movement segments according to the two-thirds power law. On average, the fit of the trajectories of monkey O did not change through practice. Monkey U showed some improvement of the fit. C. Both monkeys showed a clear improvement with practice in the fit of the predicted trajectories to the two-thirds power law compared to the beginning of practice. D. For both monkeys, the fit of the predicted trajectories to the two-thirds power law was better than the fit of the actual trajectories. The superiority in fit of the predicted trajectories increased through practice, especially for monkey O. (0.05 MB PDF) [file pcbi.1000427.s007.pdf]

**A**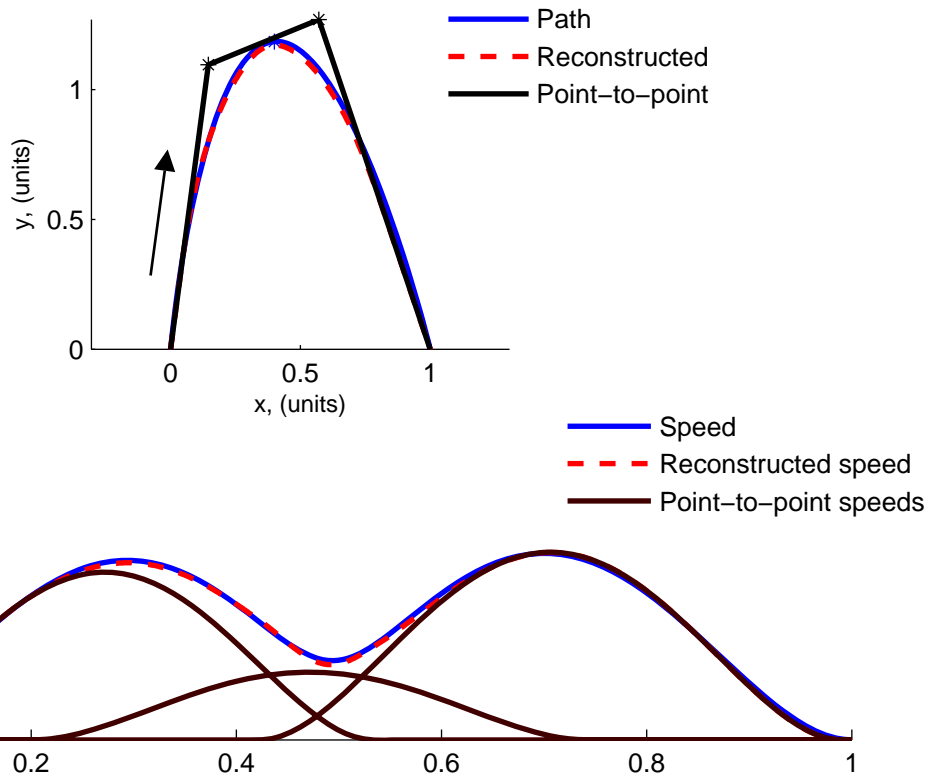**B**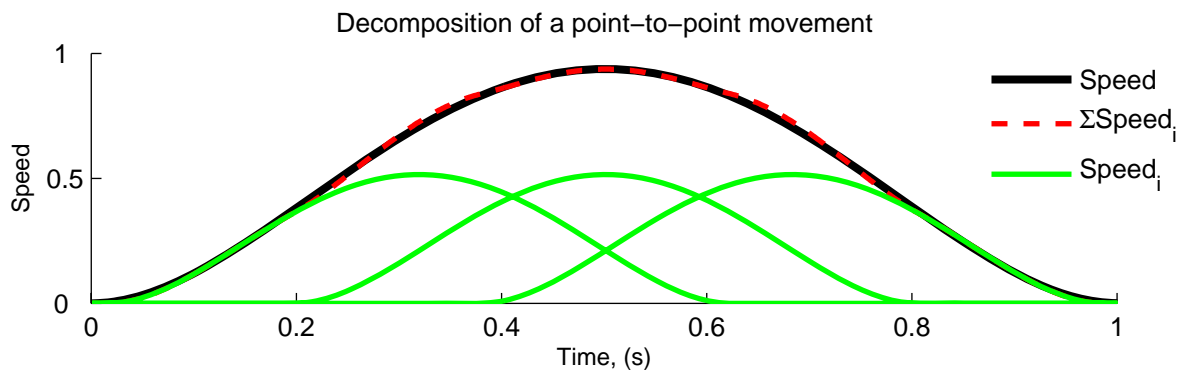

Supplement: Figure S5 — Approximation of a parabolic-like trajectory with three point-to-point movements. A (upper part). A parabolic-like path and three point-to-point movements. The approximation is marked by dashed lines. Although this result is demonstrated for a single parabolic-like trajectory, its affine transformations can be applied in case of other parabolic segments (to reconstruct parabolic-like path). A (lower part). Speed profiles of the parabolic-like trajectory (blue), approximating trajectory (dashed) and point-to-point movements. All speed profiles correspond to the respective paths from the plot above. B. Point-to-point minimum-jerk trajectory can be composed of 3 identical minimum-jerk trajectories rescaled in time and space. The ratio of peak speeds is approximately 0.55. (0.03 MB PDF) [file pcbi.1000427.s008.pdf]
