# Supplementary material for: A Compact Representation of Drawing Movements with Sequences of Parabolic Primitives
Source: PLoS Comput Biol. 2009 Jul 3;5(7):e1000427. doi: 10.1371/journal.pcbi.1000427 (PMC2699652; doi:10.1371/journal.pcbi.1000427)
Supplement: Figure S3 — An example of kinematic and equi-affine analysis for a movement segment. A. Segment path. B. A dashed-dotted graph shows the time evolution along the segment had the monkey drawn the path in A according to the constrained minimum-jerk model. C. Drawing speeds. The x axis corresponds to time for the profiles (a) and (b), and to the sample point number divided by the recording frequency, which is identical to time for actual trajectories, for profiles (a), (c). Minima and maxima of the actual and predicted trajectories occur at similar positions on the path (comparison of (a) and (c)), but their time-course is different (comparison of (b) and (c)). D. Scaled magnitudes of the regularized (with outliers omitted, see Text S1) increments of the equi-affine arc-length, scaled increments of the predicted time intervals between adjacent samples and equi-affine curvature. Several segments with an equi-affine curvature close to zero can be seen. E. Equi-affine speeds, actual and predicted (superscripts a and p respectively), were scaled to fit the same axes as in D. The predicted equi-affine speed deviates from being constant less than the actual equi-affine speed as measure γ from the formula (S8) in Text S1 indicates. (0.06 MB PDF) [file pcbi.1000427.s006.pdf]

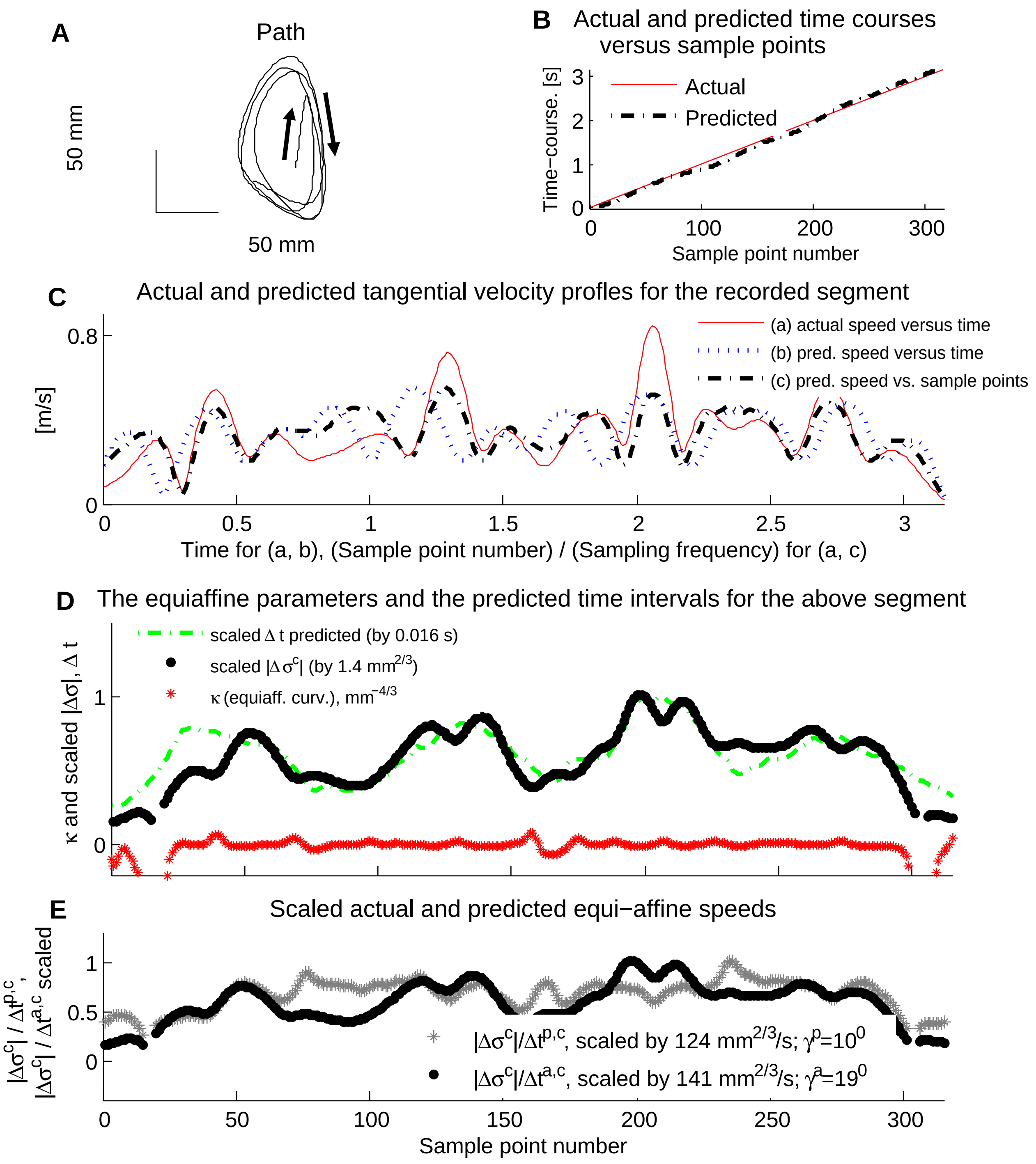

A
Path
50 mm
50 mm
B Actual and predicted time courses versus sample points
Time−course. [s]
Actual
Predicted
Sample point number
C Actual and predicted tangential velocity profles for the recorded segment
[m/s]
(a) actual speed versus time
(b) pred. speed versus time
(c) pred. speed vs. sample points
Time for (a, b), (Sample point number) / (Sampling frequency) for (a, c)
D The equiaffine parameters and the predicted time intervals for the above segment
κ and scaled |Δσ|, Δ t
scaled Δ t predicted (by 0.016 s)
scaled |Δ σ^c| (by 1.4 mm^2/3)
κ (equiaff. curv.), mm^−4/3
E Scaled actual and predicted equi−affine speeds
|Δσ^c| / Δt^p,c, |Δσ^c| / Δt^a,c scaled
|Δσ^c|/Δt^p,c, scaled by 124 mm^2/3/s; γ^p=10^0
|Δσ^c|/Δt^a,c, scaled by 141 mm^2/3/s; γ^a=19^0
Sample point number
